# Dynamic nanoassemblies derived from small-molecule homodimeric prodrugs for *in situ* drug activation and safe osteosarcoma treatment

**DOI:** 10.1016/j.isci.2023.107409

**Published:** 2023-07-17

**Authors:** Jian Wang, Peirong Xu, Yeyong Zhang, Shuai Han, Gongteng Wang, Hangxiang Wang, Haihan Song, Shufeng Li

**Affiliations:** 1 Department of Orthopedics, Shanghai Pudong New Area People’s Hospital, Shanghai, P.R. China; 2 Jinan Microecological Biomedicine Shandong Laboratory, Jinan, Shandong Province 250117, P.R. China; 3 The First Affiliated Hospital, NHC Key Laboratory of Combined Multi-Organ Transplantation, Zhejiang University School of Medicine, Hangzhou, Zhejiang Province 310003, P.R. China; 4 Department of Chemical Engineering, Zhejiang University, Hangzhou, Zhejiang Province 310027, P.R. China; 5 Department of Orthopedic Surgery, The First Affiliated Hospital of Shandong First Medical University & Shandong Provincial Qianfoshan Hospital, Shandong Key Laboratory of Rheumatic Disease and Translational Medicine, Jinan, Shandong, P.R. China; 6 Central Lab, Shanghai Key Laboratory of Pathogenic Fungi Medical Testing, Shanghai Pudong New Area People’s Hospital, Shanghai, P.R. China

**Keywords:** Medical physics, Therapeutics

## Abstract

Supramolecular prodrug self-assembly is a cost-effective and powerful approach for creating injectable anticancer nanoassemblies. Herein, we describe the self-assembly of small-molecule prodrug nanotherapeutics for tumor-restricted pharmacology that can be self-activated and independent of the exogenous stimuli. Covalent dimerization of the anticancer agent cabazitaxel via reactive oxygen species (ROS)- and esterase-activatable linkages produced the homodimeric prodrug diCTX, which was further coassembled with an ROS generator, dimeric dihydroartemisinin (diDHA). The coassembled nanoparticles were further refined in an amphiphilic matrix, making them suitable for *in vivo* administration. The ROS obtained from the coassembled diDHA synergized with intracellular esterase to activate the neighboring diCTX, which in turn induced potent cytotoxicity. In a preclinical orthotopic model of human osteosarcomas, nanoparticle administration exhibited durable antitumor efficacy. Furthermore, this smart, dual-responsive nanotherapeutic exhibited lower toxicity in animals than those of free drug combinations. We predict that this platform has great potential for further clinical translation.

## Highlights

• Homodimeric anticancer prodrugs are generated for aqueous self-assembly
• Therapeutic agents can be self-activated to achieve tumor-restricted pharmacology
• Quantitative encapsulation efficiency and high drug loading are obtained
• Nanoassemblies show effective antitumor efficacy and low toxicity *in vivo*

## Graphical abstract

**Figure:**
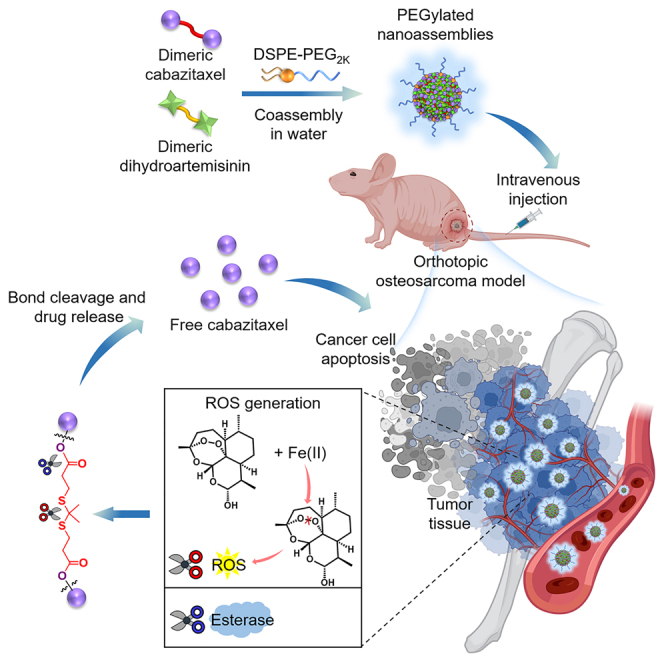

## Introduction

Supramolecular assembly of small-molecule therapeutics to form injectable nanoparticles remains a cost-effective and powerful approach to develop nanomedicines.^1^ However, most bioactive therapeutics are classified as Class II compounds within the Biopharmaceutics Classification System.^2^ They either exhibit poor water solubility or cannot be used for self-assembly without adding exogenous adjuvants. To address this, a prodrug strategy and sequential supramolecular self-assembly was developed to construct self-deliverable nanotherapies. Couvreur and colleagues chemically re-engineered therapeutics using a squalene moiety (termed squalenoylation) for the subsequent assembly of prodrugs with improved pharmacological activities.^3^^,^^4^ Dimeric prodrug nanoassemblies are alternative promising scaffolds for delivering active anticancer compounds.^5^^,^^6^^,^^7^ Ligating two therapeutic molecules with cleavable linkers generates homodimeric or heterodimeric prodrugs with altered physicochemical properties capable of self-assembling into nanoparticles. Compared with traditional matrix-based delivery systems, nanoassemblies comprising these small-molecule prodrugs have notable advantages, including: (i) exceptionally high drug loading and nearly quantitative loading efficiencies, (ii) easy generation of chemically well-defined prodrug entities, (iii) formations that are independent of excipients and obviating concerns of excipient-induced side effects, (iv) linker chemistries tailorable for target site-restricted pharmacology, (v) feasible optimization of the overall structures to improve *in vivo* performances, and (vi) simple manufacturing via self-assembly that is beneficial for scaling up.

Osteosarcoma is a common and the deadliest primary malignant bone cancer, which is prevalent in children and adolescents with an annual incidence of approximately 4.8 per million persons worldwide.^8^ It develops from primitive mesenchymal-derived bone-forming cells and usually occurs in the bone around the joints.^9^ Although radical surgical resection is the preferred choice of treatment, *en bloc* resection is difficult to attain in most cases.^10^ Chemotherapy uses toxic agents to kill cancer cells and is an important therapy for treating most cancers, including osteosarcomas. Repeated administration of chemotherapies at high doses is essential for long-term patient survival. Unfortunately, long-term medications with highly toxic chemotherapies substantially burden young patients with osteosarcoma. Some obstacles, including poor bioavailability, low accessibility to tumors, chemoresistance, and undesired drug toxicities, limit the clinical use of chemotherapy. Consequently, although most patients partially respond to this treatment, they still experience severe toxicity effects.^11^ Hence, strategies that precisely deliver more therapeutic agents to malignant tissues with manageable side effects are in high demand.

Clinically, taxane chemotherapy agents such as docetaxel have been used to treat patients with relapsed unresectable osteosarcomas.^12^^,^^13^ However, repeated administration of docetaxel results in drug resistance in patients with osteosarcoma. Cabazitaxel, a new-generation taxane, can overcome taxane resistance because of its low affinity to P-glycoprotein; however, its clinical use is impeded by its high systemic toxicity. We previously developed a thioketal (TK)-linked homogeneous dimeric cabazitaxel (diCTX) that unexpectedly demonstrated the ability for excipient-free self-assembly in aqueous solutions.^14^ However, drug activation from nanoassemblies requires light-absorbing photosensitizer and additional near-infrared (NIR) light. Upon NIR photoirradiation, the neighboring photosensitizer generates reactive oxygen species (ROS) that trigger TK bond cleavage and subsequently activate the cabazitaxel agent.^15^ Unfortunately, limited tissue penetration makes the efficient delivery of NIR light to deep-seated cancers somewhat difficult in clinical practice. We therefore hypothesized that self-activating nanosystems that do not require exogenous light irradiation are beneficial for treating inaccessible cancers.

**Figure 1:**
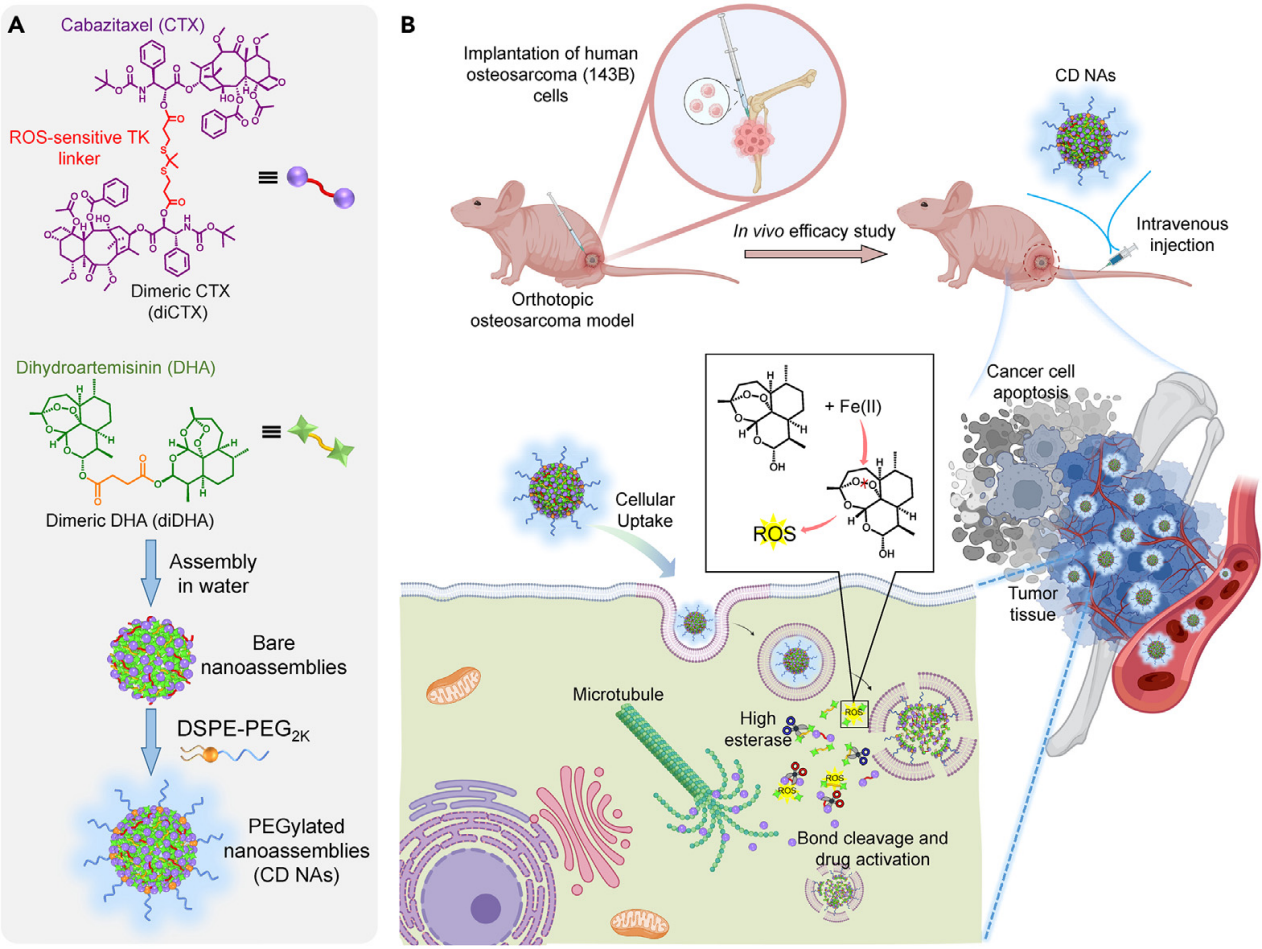
Schematic of supramolecular nanoassembly, intravenous administration, and reactive oxygen species (ROS)-responsive drug activation for osteosarcoma chemotherapy (A) Chemical structures of the dimeric cabazitaxel (diCTX) and dimeric dihydroartemisinin (diDHA) used in this study. Self-assembly of both dimeric prodrugs into nanostructures in aqueous solutions. Coassembling the nanoparticles with a DSPE-PEG_2k_ matrix was performed to PEGylate the surfaces. (B) Schematic showing the efficacy of using CD nanoassemblies against orthotopic osteosarcoma in mice. Once taken up by the osteosarcoma cells, ROS are generated by the codelivered diDHA, which subsequently triggers the activation of cabazitaxel from diCTX. Intracellular esterase also is expected to activate cabazitaxel by hydrolyzing the ester bond in diCTX. Illustration created with BioRender.com.

Based on this hypothesis, we report that the nanoassembly of homodimeric cabazitaxel prodrug can be activated *in situ* by endogenously generated ROS in cancer cells in the absence of exogenous photosensitizers. Our approach is based on the homogeneous linking of anticancer drug cabazitaxel via TK linker into a single self-assembled chemical entity that could subsequently coassemble with the dimeric ROS generator dihydroartemisinin (DHA) (Figure 1A). The prodrug nanoassemblies (termed CD nanoassemblies) were stabilized through multiple intermolecular interactions. The particle surfaces were further PEGylated with 1,2-distearoyl-*sn*-glycero-3-phosphoethanolamine-*N*-[methoxy (polyethylene glycol)2000] (DSPE-PEG_2k_) at a low weight ratio (10%) to extend the blood circulation after intravenous injection. Once taken up by the cancer cells, the ROS produced by the neighboring DHA cleave the TK bonds to release pharmacologically active cabazitaxel (Figure 1B). Furthermore, in a preclinical orthotropic model of aggressive human osteosarcomas, CD nanoassemblies increased the drug accumulation at the tumor sites, along with durable tumor regression effects and low systemic toxicities.

## Results

### Rational design of self-activating nanoassemblies comprising dimeric prodrugs

The successful nanoassembly of hydrophobic diCTX in aqueous solutions indicated that this strategy could be extended to other therapeutic molecules such as DHA. DHA is a derivative and the main active metabolite of artemisinin.^16^ Artemisinin was originally isolated from the Chinese medicinal herb *Artemisia annua* and is recommended as a first-line treatment for malaria by the World Health Organization. One of the most widely accepted antimalarial mechanisms is that endoperoxide-containing DHA reacts with Fe^2+^ ions to produce free radicals (Figure 1B).^17^^,^^18^ To render DHA capable of self-assembly, we synthesized a homogeneous DHA dimer (diDHA) ligated by a non-cleavable succinic acid linker. TK-linked diCTX was synthesized as previously described.^14^ The dimeric compound syntheses were unambiguously confirmed by NMR spectra (e.g., ^1^H and ^13^C NMR, Figures S1–S4). In addition, the purities of both prodrugs were characterized by the high-performance liquid chromatography (HPLC; Figure S5).

### Coassembly of dimeric prodrugs in aqueous solution and morphological characterization of nanoparticles

**Figure 2:**
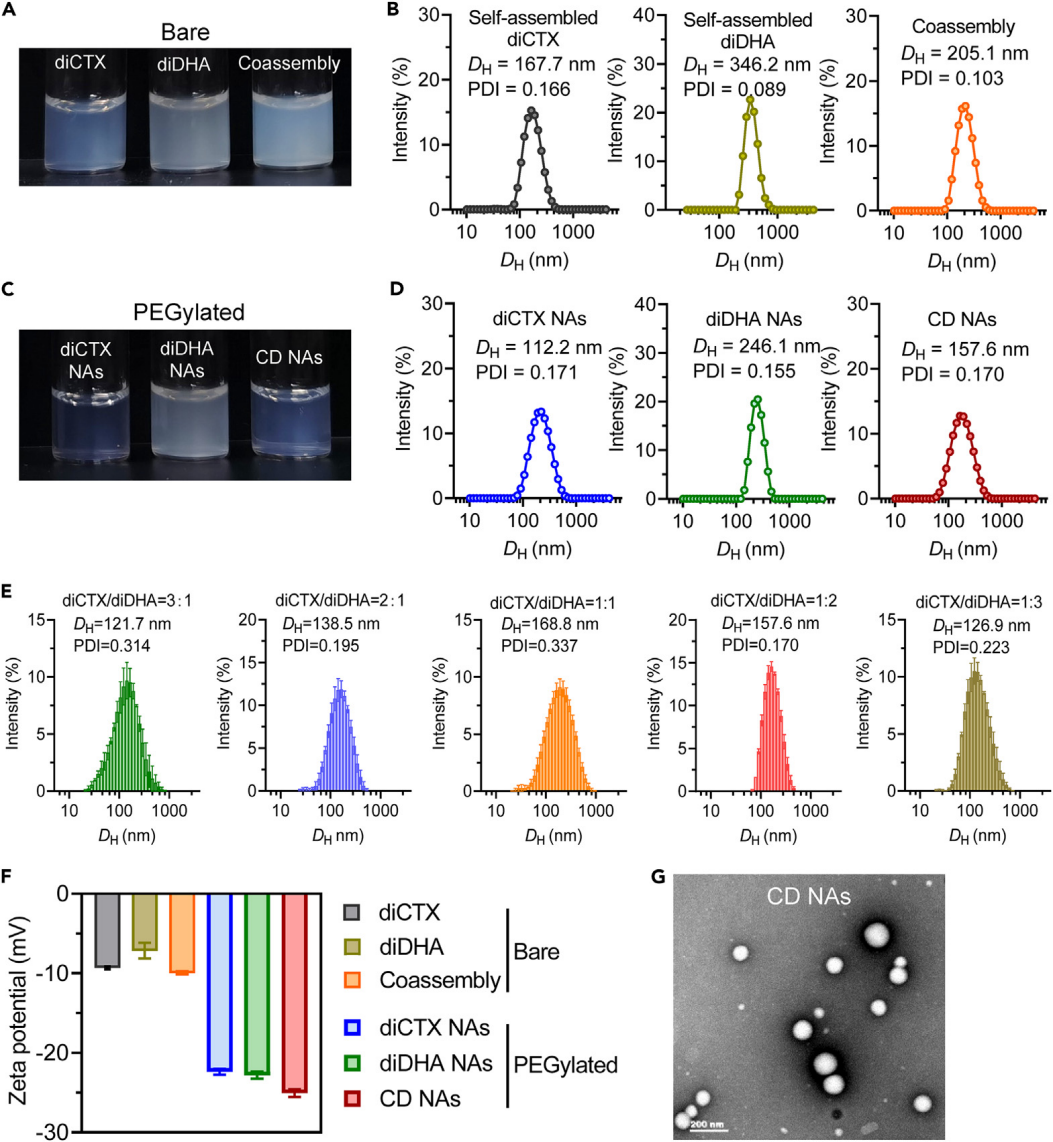
Characterization of nanoassemblies constructed from diCTX and diDHA (A and B) (A) Photograph and (B) dynamic light scattering (DLS) analyses of the bare nanoassemblies without PEGylation. (C and D) (C) Photograph and (D) DLS analyses of the PEGylated nanoassemblies. (E) DLS measurements of nanoparticles coassembled from diCTX and diDHA prodrugs at varying molecular ratios. (F) Zeta potentials of individual nanoassemblies and coassembled nanoparticles. Data are presented as the mean ± standard deviation (n = 3). (G) Representative transmission electron microscopy image of CD nanoassemblies. Scale bar, 200 nm.

After synthesizing these homodimeric prodrugs, we tested their self-assembly in aqueous solutions. We followed a reprecipitation protocol, where solutions of individual prodrugs or prodrug mixtures in DMSO were injected into deionized (DI) water to form stable colloidal nanosuspensions (Figure 2A). Dynamic light scattering (DLS) analysis confirmed the formation of the nanostructures with narrow monomodal distribution (Figure 2B). Surface cloaking by PEGylation not only increases the solubility and stability of products but is also expected to reduce clearance from the body by the mononuclear phagocyte system after systemic administration. Therefore, the coassembled prodrug particle surfaces were further PEGylated with DSPE-PEG_2k_ at a 10% weight ratio to produce systemically injectable solutions (Figures 2C and 2D). Interestingly, both prodrugs were miscible with each other at arbitrary molar ratios, as measured by DLS (Figure 2E). The nanoparticles prepared from diCTX and diDHA were designated as CD nanoassemblies. The surface charges of the PEGylated nanoassemblies were negative because of the presence of negative DSPE-PEG_2k_ matrices (Figure 2F), which also increased the particle stability. The resulting CD nanoassemblies were further characterized using transmission electron microscopy (TEM), which revealed the formation of uniform spherical nanostructures (Figure 2G). Conversely, chemically unmodified DHA was incapable of self-assembly using the same protocol (Figure S6A). Furthermore, while free DHA was assembled into the hydrophobic core of the diCTX nanoassemblies, the particles spontaneously precipitated after 12 h (Figure S6B). The encapsulation efficiencies and drug loading contents (DLCs) of the CD nanoassemblies are summarized in Table S1. Nearly quantitative self-assembly was achieved, with an exceptionally high total DLC reaching 88.0%.

### Aqueous stability and stimuli-responsive drug activation

**Figure 3:**
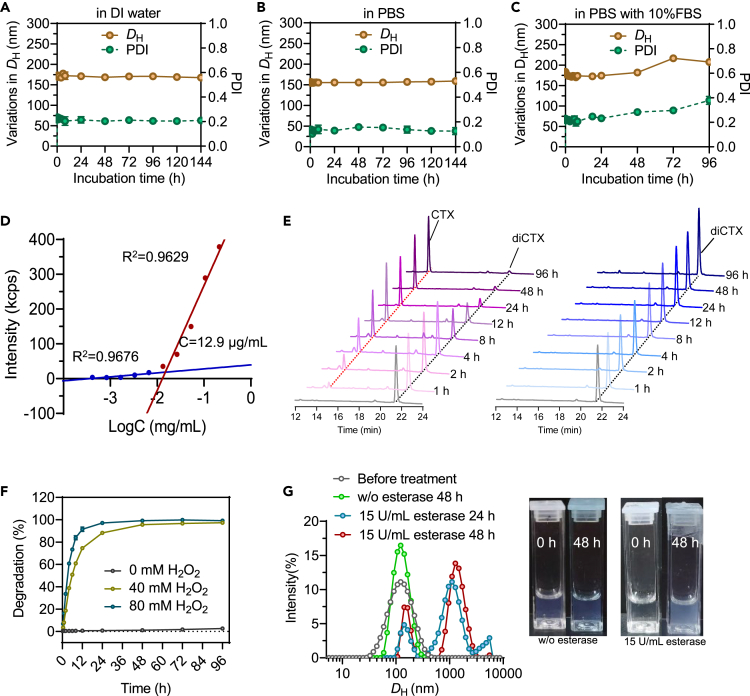
*In vitro* characterization of CD nanoassemblies (A–C) Stability of the CD nanoassemblies in DI water, PBS (pH 7.4), and PBS containing 10% (v/v) fetal bovine serum, as verified by changes in particle size (*D*_H_) and polydispersity index (PDI). (D) CMCs of the CD nanoassemblies, as determined by the pyrene method. (E and F) (E) Representative HPLC chromatograms of the diCTX prodrug in the presence and absence of H_2_O_2_ and (F) quantification of free cabazitaxel release under different conditions. (G) Variations in size distribution of nanoassemblies after incubations with esterase. Insets: photographs of the particle solutions before and after the treatments with esterase.

Stability testing suggested a relatively low stability of the bare CD nanoassemblies (Figure S7); however, surface PEGylation increased the colloidal stability of these nanoassemblies (Figures 3A–3C). For different aqueous media, including DI water, PBS, and PBS containing 10% (v/v) fetal bovine serum, neither significant variations in diameter nor precipitates were observed over a four-day observation (Figures 3A–3C). Furthermore, when circulating in the blood, nanoassemblies that rely on noncovalent interactions tend to disassemble into free molecules below the critical micelle concentration (CMC). Therefore, CMC is an important parameter for designing drug delivery vehicles. Consequently, we determined the CMCs of the CD nanoassemblies to be 12.9 μg/mL (Figure 3D). This CMC was lower than those of the micelles formed from DSPE-PEG_2K_ (approximately 30 μg/mL). The low CMC of the nanoparticles could contribute to their thermodynamic stability and high structural integrity during blood circulation.

To study whether active cabazitaxel can be released in response to ROS, we examined the drug activation profiles after exposing the diCTX prodrug to hydrogen peroxide (H_2_O_2_). After a 2-h incubation with H_2_O_2_, a distinct peak consistent with free cabazitaxel was observed in the HPLC spectra (Figure 3E), suggesting that the primary species released was chemically unmodified cabazitaxel. The release of the free drug was rapid, with approximately 91.4% releases after incubating for 12 h with 80 mM H_2_O_2_ (Figure 3F). In contrast, the diCTX prodrug remained stable and free cabazitaxel was not released in the absence of H_2_O_2_ (Figures 3E and 3F). In addition, variations in particle size distribution in response to esterase were observed in aqueous solution by DLS measurements. After incubation with esterase, large particles and aggregates formed, which could be ascribed to esterase-responsive drug activation and structural disruption (Figure 3G). These results suggest that not only is the TK bond within the cabazitaxel prodrug susceptible to ROS and can be exploited for triggered drug activation but also this class of dimer conjugates remains sufficiently stable in the blood circulation.

### Molecular dynamics simulations

**Figure 4:**
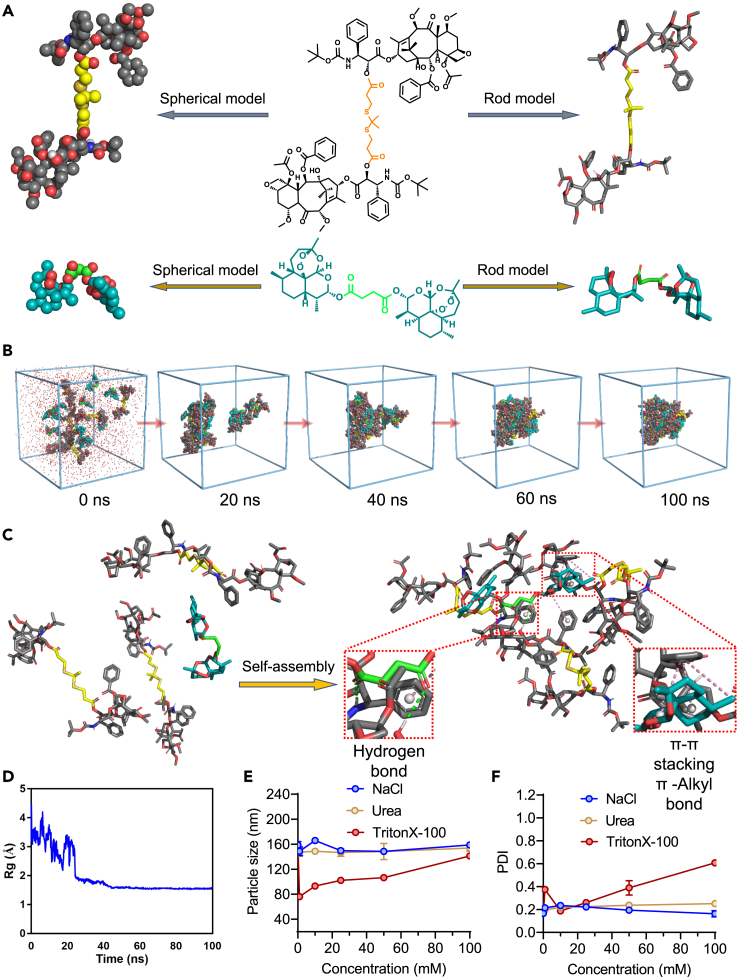
Molecular dynamics (MD) simulations of the self-assembly of CD nanoassemblies and exploration of molecular interactions (A) Chemical structures and molecular models of the diCTX and diDHA prodrugs. (B) MD simulations over 100 ns. The initial snapshot includes six diCTX and twelve diDHA molecules that were randomly dispersed. Self-aggregation occurred upon simulation. The snapshot after 100 ns showed the generation of a stable compact nanoparticle. (C) Multiple noncovalent interactions in the nanoassemblies, indicated by pink (π–π stacking and π–alkyl bond) and green (hydrogen bond) dashed lines. (D) The radius of gyration (Rg) decreased gradually over the course of the simulation, suggesting a tendency to aggregate. (E and F) DLS measurements of CD nanoassemblies after incubation with sodium chloride, urea, and Triton X-100 (i.e., 0, 1, 10, 25, 50, and 100 mM) showed the changes in (E) particle size and (F) PDI. Data are presented as the mean ± standard deviation (n = 3).

Molecular dynamics (MD) simulations were performed to gain insights into the mechanisms of self-assembly of these hydrophobic dimer prodrugs (Figure 4A). The diCTX and diDHA molecules were placed in a cuboid box with explicit water and subjected to 100 ns of MD simulations. At the beginning of each simulation, eighteen molecules were randomly dispersed and separated from each other in water (Figure 4B). They spontaneously self-aggregated over the course of the simulation and eventually formed a compact nanostructure at 60 ns. A stable aggregated state was captured after approximately 60 ns, with no free molecules released from the nanoassembly over the entire simulation (100 ns). We further explored the possible molecular interactions involved in the assembly. Typically, π–π stacking, van der Waals interactions, and hydrogen bonds favor nanoparticle formation (Figures 4C and 4D). Overall, these intramolecular and intermolecular interactions drive the nanoassembly of the prodrugs and contribute to the stability of the entire nanosystem. Our simulations supported the TEM and DLS experimental finding that hydrophobic dimers were capable of self-assembling in aqueous media.

Sodium chloride, urea, and Triton X-100 are known to attenuate electrostatic interactions, hydrogen bonding, and hydrophobic interactions, respectively. We thus tested their disruptive effects on the nanoassemblies. Adding the surfactant Triton X-100 (i.e., 0, 1, 10, 25, 50, and 100 mM) gradually dissociated the nanoassemblies, presumably due to hydrophobic competition; however, sodium chloride and urea changed neither the particle size nor the polydispersity index (Figures 4E and 4F). These data suggest that hydrophobic interactions significantly contributed to the diCTX and diDHA prodrug coassembly, with hydrogen bonds also participating in the particle formation.

### *In vitro* cytotoxicity against osteosarcoma cancer cells and normal cells

**Figure 5:**
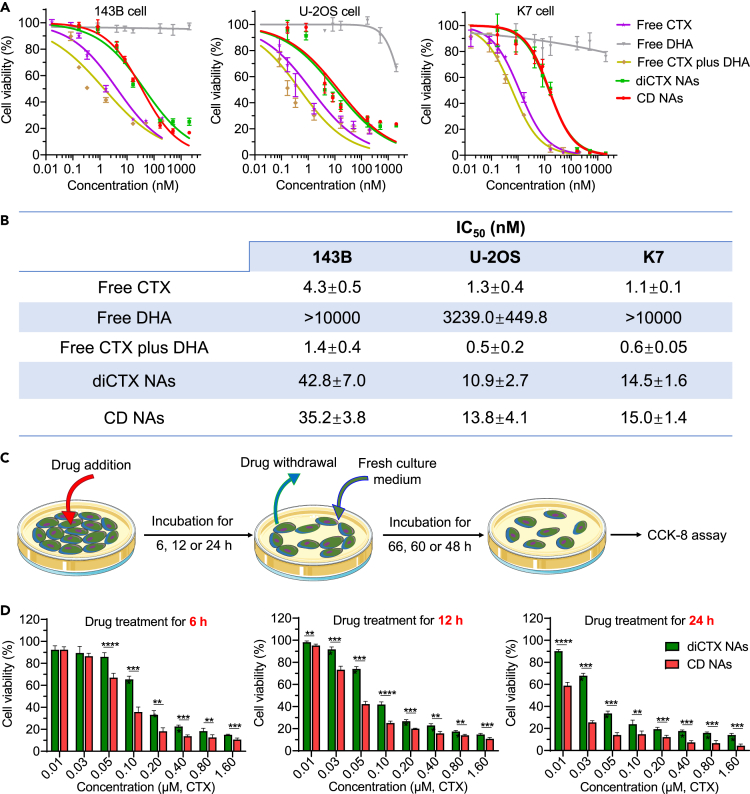
CD nanoassemblies (NAs) showed potent cytotoxicity against osteosarcoma cells (A) *In vitro* cytotoxicity of various drugs in cancer cells. The cells were exposed to the drugs for 72 h and cell viability was determined using CCK-8 assays. Data are presented as the mean ± standard deviation (SD; n = 3). (B) Half maximal inhibitory concentrations (IC_50_) extrapolated from the dose-response curves. (C) Schematic illustration of the experimental procedure for drug withdrawal. 143B cells were treated with each drug for 6, 12, or 24 h and subsequently incubated with fresh media for additional 66, 60, or 48 h, respectively. (D) Viability of the 143B cells after exposure to each drug for 6, 12, and 24 h, as determined by the CCK-8 assays. Data are presented as the mean ± SD (n = 5). ∗p < 0.05, ∗∗p < 0.01, ∗∗∗p < 0.001, and ∗∗∗∗p < 0.0001.

We next assessed the cytotoxicity of the nanotherapeutics against osteosarcoma-derived cancer cells using a cell counting kit-8 (CCK-8) assay. Several human osteosarcoma cell lines, including 143B, U-2OS, and K7, were included in this experiment and treated with different drug concentrations. After 72 h, the cell viabilities were determined using CCK-8 assays, and the half maximal inhibitory concentrations (IC_50_) were extrapolated from the dose-response curves. The data are presented in Figures 5A and 5B. As expected, the free DHA was negligibly active, with almost no cytotoxicity within the tested concentrations. Free cabazitaxel had high cytotoxicity with IC_50_ values in the nanomolar range, suggesting the potency of cabazitaxel in inducing osteosarcoma cell apoptosis. After ligating cabazitaxel into dimeric entities via TK linkages and sequential nanoassembly, the *in vitro* potency of the diCTX nanoassemblies was reduced compared to that of free cabazitaxel, which could be due to the delayed activation of cabazitaxel from the nanoassemblies. Moreover, we found that irrespective of the diDHA coassembly, the CD nanoassemblies exhibited IC_50_ values that were comparable with those of the diCTX nanoassemblies (Figure 5B). Strikingly, the cytotoxicity of CD nanoassemblies was reduced in noncancerous cells; for example, compared with free drug combination, relatively less cytotoxicity was observed for CD nanoassemblies across tested human umbilical vein endothelial cells and RAW 264.7 mouse macrophage cells (Figure S8 and Table S2).

To further examine whether integrating an ROS generator such as diDHA could improve the cytotoxicity of the dimeric cabazitaxel compared with that of the diCTX nanoassembly monotherapy, we performed an additional *in vitro* cytotoxicity analysis (Figure 5C). After exposing 143B cells to nanoassemblies for different durations (6, 12, and 24 h), the drug-containing cell culture media were replaced with fresh culture media. Following an additional incubation, the cell viability was determined (Figure 5D). Interestingly, we observed that the inhibitory effects of the CD nanoassemblies were superior to those of the diCTX nanoassemblies alone for each tested treatment duration. This difference may be due to the additional incorporation of the diDHA agents into the nanoassemblies. Considering the non-cytotoxicity of diDHA at these concentrations, the augmented cytotoxicity could be attributed to a bystander effect of diDHA in activating cabazitaxel through its ROS-generating capacity.

### Cellular uptake of nanoassemblies and intracellular ROS generation

**Figure 6:**
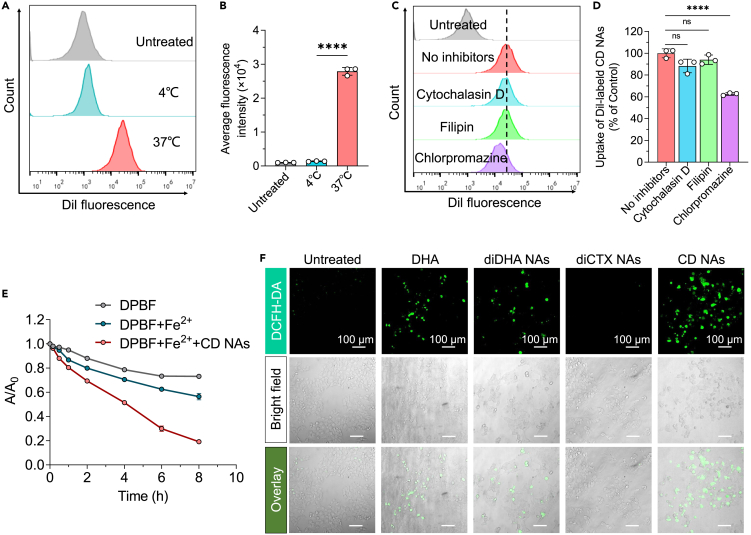
Clathrin-dependent endocytosis of CD nanoassemblies (NAs) and ROS production detected by the ROS fluorescence probes (A and B) Cellular uptake of DiI-labeled CD nanoassemblies after incubating at 37°C or 4°C using flow cytometry analysis. Data are presented as the mean ± standard deviation (SD; n = 3); ∗∗∗∗p < 0.0001. (C and D) Cellular uptake in the presence or absence of three inhibitors that affect distinct endocytic pathways. The cells were pretreated with the inhibitors, and then were combined with the DiI-labeled nanoassemblies for 4 h. Data are presented as the mean ± SD (n = 3); ns, not significant; ∗∗∗∗p < 0.0001. (E) ROS generation in the presence of Fe^2+^/CD nanoassemblies detected by the ROS fluorescence probe 1,3-diphenylisobenzofuran. Data are presented as the mean ± SD (n = 3). (F) Fluorescence images showing the intracellular ROS production, as detected by the ROS fluorescence probe 2′,7′-dichlorofluorescein diacetate (DCFHDA). Cells were treated with various drugs before adding DCFHDA. The fluorescence signals were observed using a fluorescence microscope.

To examine whether active transport dominates the cellular uptake of the CD nanoassemblies, nanoassemblies labeled with the lipophilic fluorescent dye DiI were incubated with 143B cells at different temperatures for 4 h. Compared with the prompt uptake observed at 37°C, lowering the temperature to 4°C significantly impaired the internalization of the nanoparticles, suggesting energy-dependent endocytosis (Figures 6A and 6B). We further assessed the specific endocytic pathways of the CD nanoassemblies. The cells were pretreated for 30 min with three inhibitors that affected distinct pathways, and then incubated with dye-labeled nanoassemblies for another 4 h. As shown in Figures 6C and 6D, chlorpromazine, a chemical inhibitor of clathrin-mediated endocytosis, markedly reduced the particle uptake to 63.5%. In contrast, cytochalasin D and filipin (inhibitors of pinocytosis and caveolin-dependent endocytosis, respectively) did not affect the internalization of the nanoassemblies. These results demonstrate that clathrin-mediated endocytosis is the prominent pathway for CD nanoassemblies.

We further explored whether coassembled diDHA can generate ROS to activate cabazitaxel via ROS-triggered TK bond cleavage and impart cytotoxic damage to the cells, eventually inducing cell apoptosis. Before the intracellular assessment, we examined the ROS generation capabilities of the CD nanoassemblies in test tubes using 1,3-diphenylisobenzofuran (DPBF) as the ROS probe. Compared with moderate changes in the DPBF absorbance spectra, adding Fe^2+^/CD nanoassemblies substantially diminished the DPBF absorbance (Figure 6E). Furthermore, intracellular ROS generation was detected using the fluorescence probe, 2′,7′-dichlorodihydrofluorescein diacetate, which can be oxidized by ROS and converted into dichlorofluorescein to emit green fluorescence. Interestingly, green fluorescence was detectable in 143B cells pretreated with DHA (Figure 6F). Notably, treatment with the CD nanoassemblies led to more efficient ROS generation in cells, indicated by bright green signals, presumably due to the elevated uptake of DHA by the nanodelivery. In contrast, the cells without DHA treatment showed very weak fluorescence (Figure 6F). These results were consistent with previous results and demonstrated that ROS production may contribute to the improved potency of cabazitaxel in cancer cells.^19^

### *In vitro* antiproliferation and apoptosis-inducing capacity

**Figure 7:**
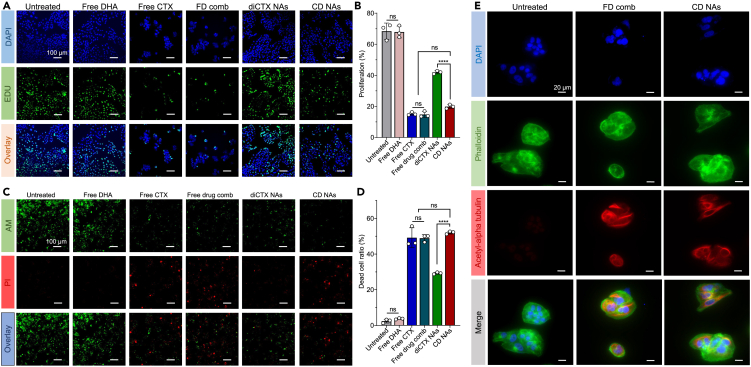
Examination of the cytotoxic effects of CD nanoassemblies (NAs) on 143B cells. A drug combination (FD comb) was also included for comparison (A) Representative confocal microscopy images to determine cell proliferation. A Click-iT EdU assay quantified the cancer cell proliferation. The cells were treated with CD nanoassemblies (at a 30 nM cabazitaxel equivalent concentration) for 48 h. Untreated cells were used as the controls. (B) Quantification of EdU-positive cells. Data are expressed as the mean ± standard deviation (SD; n = 3); ns, not significant; ∗∗∗∗p < 0.0001. (C) Calcein-AM/propidium iodide (PI) double-staining assay used to quantitatively analyze the live and dead cells after the drug treatment. The calcein-AM and signal PI signals of the same field were captured at 488 (green) and 545 nm (red), respectively, and then merged to produce new fluorescence images. (D) Quantification of dead cell ratios. Data are expressed as the mean ± SD (n = 3); ns, not significant; ∗∗∗∗p < 0.0001. (E) Cabazitaxel-induced cell morphology changes and microtubule bundle formation (red), F-actin (green), and nucleus (blue).

We used a 5-ethynyl-2′-deoxyuridine (EdU) incorporation assay to evaluate the antiproliferation activity and further examine the apoptosis induced by the CD nanoassemblies. EdU incorporates into DNA during the synthesis phase of the cell cycle, thus labeling cells that undergo proliferation.^20^ After exposing 143B cancer cells to each drug for 48 h, fluorescence cell imaging was performed. As shown in Figures 7A and 7B, DHA showed no antiproliferation capacity in the cells, with cell proliferation rates comparable with those of untreated cells (p > 0.05 versus the untreated control group). In sharp contrast, free cabazitaxel and the free drug combination significantly reduced cell proliferation. The CD nanoassemblies were also effective, with decreased cell numbers and diminished proliferation rates. No statistical differences in the proliferation rates were observed between the CD nanoassembly-treated and free drug combination–treated groups. Interestingly, the diCTX nanoassemblies alone exhibited significantly reduced antiproliferation activities compared with the CD nanoassemblies, suggesting that diDHA indeed played a bystander role in augmenting the cytotoxicity of diCTX.

A live/dead cell assay was performed to visually assess the viability of the 143B cells after the CD nanoassembly treatments. Dual staining with calcein-AM and propidium iodide (PI) was used to assess the activities by counting the live and dead cells. Calcein-AM stains viable cells and emits green fluorescence, while PI only enters dead cells and produces red fluorescence. This method readily distinguishes apoptotic cells (red fluorescence) from live cells (green fluorescence). As shown in Figures 7C and 7D, the free DHA was non-cytotoxic, with cell death rates comparable with those of untreated cells. As expected, the 143B cells treated with the CD nanoassemblies predominantly fluoresced red, indicating a significant induction of apoptosis. Again, we confirmed that the CD nanoassemblies had a cell killing activity superior to that of the diCTX monotherapy. Therefore, compared with free cabazitaxel or the free drug combination, the *in vitro* cytotoxicity of the diCTX-derived nanoassemblies was lower. This could be due to the delayed activation of free cabazitaxel from the nanoassemblies. Fortunately, coassembly with a dimeric ROS-generating agent successfully rescued the activity of diCTX. Taken together, these *in vitro* data indicate that coassembled diDHA specifically triggered ROS generation after cellular uptake, thereby partially overcoming the low conversion of self-assembled dimeric cabazitaxel prodrugs.

Cabazitaxel binds to β-tubulin subunits and promotes microtubule stabilization, which eventually induces multinucleation and the apoptosis of rapidly proliferating cancer cells. To examine whether the observed cytotoxicity was associated with the released cabazitaxel, we immunostained the microtubules in the 143B cells. On exposing the cells to the CD nanoassemblies, the microtubules, nuclei, and F-actin were fluorescently labeled with an acetylated α-tubulin antibody (red), 4′,6-diamidino-2-phenylindole (blue), and phalloidin (green), respectively. Confocal laser scanning microscopy (CLSM) revealed that cells underwent multinucleation and polymerization of microtubules after exposure to free cabazitaxel or CD nanoassemblies (Figure 7E). However, the untreated cells showed no such morphological changes. Therefore, the cytotoxicity of the CD nanoassemblies could be attributed to the released cabazitaxel, which was consistent with the results of the ROS-responsive release of chemically unmodified cabazitaxel.

### Nanodelivery increased intratumoral accumulation in a mouse model of orthotopic osteosarcoma

**Figure 8:**
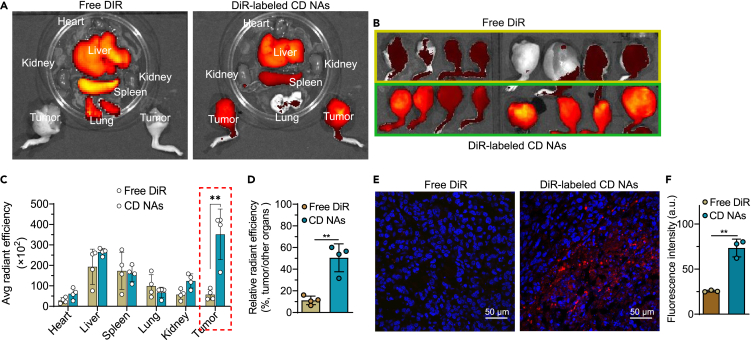
Tissue biodistribution and intratumoral drug delivery in an orthotopic 143B osteosarcoma model established in the tibias of mice (A and B) *Ex vivo* fluorescence images of the major organs (heart, liver, spleen, lung, and kidneys) and tumors 24 h post-administration. To track the *in vivo* distributions of the CD nanoassemblies, the lipophilic near-infrared (NIR) dye 1,1′-dioctadecyl-3,3,3′,3′-tetramethylindotricarbocyanine iodide (DiR) was coassembled with the CD nanoassemblies. Free DiR and DiR-labeled CD nanoassemblies were injected at 20 μg DiR equivalent doses per mouse via the tail vein. Data are presented as the mean ± standard deviation (SD) (n = 4). ∗∗, p < 0.01. (C) Quantitative analysis of the organ fluorescence intensities. (D) Quantitative analysis of the fluorescence fractions of the tumors to other major tissues. (E and F) (E) Representative CLSM images and (F) fluorescence intensities of tumor tissue sections excised 24 h post-administration (Scale bars, 50 μm). Data are presented as the mean ± SD (n = 3). ∗∗, p < 0.01.

Enhanced tumor accumulation is indispensable for a favorable *in vivo* efficacy and reduced side effects of therapeutic nanoparticles. To show the localization of the administered nanoassemblies, we first established a clinically relevant osteosarcoma model. Immunodeficient BALB/c nude mice were orthotopically implanted with 143B cancer cells. The CD nanoassemblies were noncovalently labeled with the NIR fluorescent dye 1,1′-dioctadecyl-3,3,3′,3′-tetramethylindotricarbocyanine iodide (DiR) and intravenously injected into the mice. At 24 h post-administration, the mice were euthanized and their major organs and tumors excised for *ex vivo* NIR fluorescence imaging (Figures 8A and 8B). The NIR imaging results showed an accumulation of free DiR primarily in the livers and spleens but not in the tumors. Treatment with the CD nanoassemblies resulted in strong fluorescence from the tumors, although the same was observed for the livers and spleens. Further quantitative analysis showed that the fluorescence intensities of the mouse tumors treated with the CD nanoassemblies were 6.2-fold higher than those treated with free DiR (Figure 8C). In addition, the fluorescence fractions of the tumors to the other major tissues were 50.5% ± 12.9% and 11.2% ± 4.0% for the mice administered with CD nanoassemblies and free DiR, respectively (Figure 8D).

The tumor tissues were cryosectioned and the drug accumulation was examined by CLSM. The CD nanoassemblies had a higher intratumor delivery than free DiR, as evidenced by the extensive red fluorescence signals in the tumor cross-sections (Figure 8E). Consistently, higher fluorescence intensities were observed in the CD nanoassembly-treated mice, whereas the mice administered with free DiR showed negligible fluorescence (Figure 8F). These data suggest that the covalent ligation of small-molecule therapeutics into homodimeric prodrugs followed by self-assembly is an efficient approach to augment drug delivery to target tumor lesions, which could potentiate the subsequent treatment efficacy and reduce drug toxicity.

### Nanoassemblies suppresses tumor growth against orthotopic 143B osteosarcomas without inducing systemic toxicity

**Figure 9:**
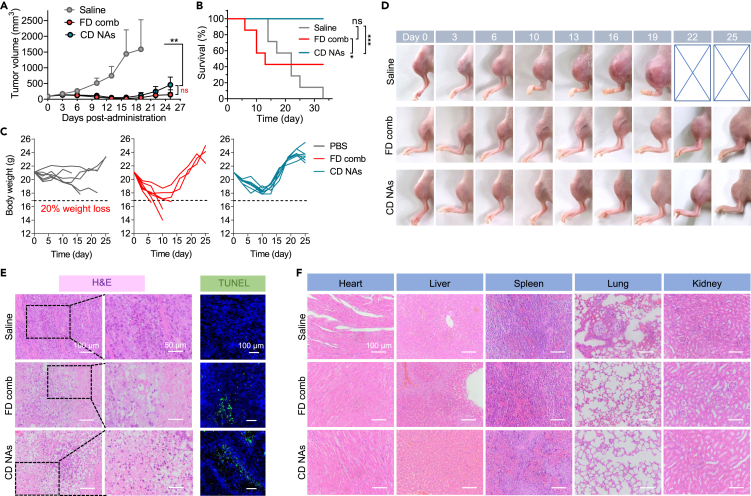
Therapeutic efficacy of CD nanoassemblies (NAs) against an orthotopic 143B osteosarcoma-bearing BALB/c nude mouse model A drug combination (FD comb) was also included for comparison. (A and B) (A) Tumor growth curves and (B) mouse survival after treatment. Data are presented as the mean ± standard deviation (n = 7). ∗, p < 0.05; ∗∗, p < 0.01; ∗∗∗, p < 0.001. (C) Individual body weight of the mice after different treatments. (D) Photographs of the 143B tumors from each treatment group during the observation. (E and F) Histological analysis of tumor tissues (E) and major organs (F) after different treatments.

Due to the clinical therapeutic interest in cabazitaxel, we finally assessed the efficacy of the CD nanoassemblies in a clinically relevant orthotopic osteosarcoma model. We implanted 143B cancer cells in the tibias of immunodeficient BALB/c nude mice to establish orthotopic xenograft models. When the tumors reached approximately 100 mm^3^ in volume, the mice were administered the CD nanoassemblies at a dose of 7.0 mg/kg cabazitaxel and 4.8 mg/kg DHA in three-day intervals in the nanoparticle or soluble form. The orthotopic osteosarcomas were aggressive, with the average tumor volume reaching approximately 2000 mm^3^ after 22 days in the saline-treated group (Figures 9A and 9D). Free cabazitaxel combined with DHA generated potent tumor inhibition, suggesting the efficacy of free cabazitaxel in this osteosarcoma model (Figure 9A). However, we observed dramatic mouse deaths and loss of body weight in the animals that received a combination of free cabazitaxel and DHA (Figure 9C). The administration of the CD nanoassemblies (prodrug/DSPE-PEG_2k_, 10:1, w/w) suppressed considerable tumor growth compared with saline treatments (p < 0.01) and had tumor growth efficiencies comparable with those of the free drug combination (p > 0.05). Strikingly, the mouse survival after the CD nanoassembly treatment was noticeably improved, with all mice surviving the observation period (Figure 9B). In addition, the body weights of the mice receiving this dose slightly decreased but quickly rebounded to normal after the cessation of treatment, indicating that this regimen was well tolerated in animals. H&E staining and terminal deoxynucleotidyl transferase-mediated dUTP-biotin nick end labeling staining also verified the extensive intratumoral apoptosis following the CD nanoassembly treatment (Figure 9E). These histological analyses are consistent with the tumor inhibition results. H&E staining of major organs also revealed no evident damage in mice after the treatments of free drug combination CD nanoassemblies (Figure 9F).

## Discussion

The development of high-efficiency and low-toxic treatment modalities for patients with osteosarcoma is urgently required. Cabazitaxel is a chemotherapy in the taxane family with a high *in vitro* potency through mitotic arrest at the metaphase-anaphase transition.^21^^,^^22^ Importantly, this drug can overcome the resistance induced by taxanes. So far, a clinical formulation (Jevtana^Ⓡ^, Sanofi-Aventis) in polysorbate 80 with a 13% ethanol aqueous solution has been approved by the U.S. Food and Drug Administration for treating metastatic castration-resistant prostate cancer (mCRPC) patients that are refractory to docetaxel. While, Jevtana^Ⓡ^ has extended the survival of mCRPC patients in clinical trials,^23^ this regimen has shown a high dose-limiting toxicity with a maximum tolerated dose of only 25 mg/m^2^ for a single injection, which shows a substantially higher toxicity than those of paclitaxel (175 mg/m^2^) and docetaxel (75 mg/m^2^).^24^ To address these issues, some formulation strategies such as polymeric conjugation, polyunsaturated fatty acids, and dimerization-induced self-assembly have been developed.^25^^,^^26^^,^^27^^,^^28^^,^^29^^,^^30^ Among these, dimeric prodrug-based nanoassemblies were of particular interest because of their capacity for high drug loading, easy chemical synthesis, and feasible manufacture for injectable nanoparticles.^14^^,^^31^

When circulating in the blood, these prodrug nanoassemblies remain biologically inactive and maintain their structural integrity to inhibit the premature release of the drug payloads. When accumulated in the tumor lesion or taken up by cancerous cells, the chemical bonds in the dimeric prodrugs are susceptible to endogenous stimuli and may undergo cleavage to release the active drugs. Herein, a TK linker with an ROS and esterase dual responsivity was devised to create self-assembling prodrug entities. Furthermore, compared with conventional ROS generation methods that combine a photosensitizer with NIR irradiation, we showed that a codelivered DHA dimer may act as an ROS generator, which could exert a bystander effect to trigger the activation of nearby cabazitaxel agents. The present strategy does not require NIR irradiation for ROS generation, making it possible to treat deep-seated cancer. In cell-based *in vitro* experiments, we observed that DHA codelivery potentiated the cytotoxicity of the diCTX nanoassemblies alone. Further *in vivo* studies revealed that the CD nanoassemblies facilitated notable intratumor accumulation after being systemically administered. The therapeutic activities of the nanoassemblies were evaluated in a preclinical orthotopic osteosarcoma model. Compared with free cabazitaxel administration, the CD nanoassemblies demonstrated substantial tumor repression efficacies with less induced toxicity.

In summary, by exploiting the homogeneous dimerization of hydrophobic drugs and sequential nanoassembly, we have developed a self-assembling nanotherapeutic strategy for the high-efficiency loading of structurally dissimilar prodrug cocktails. Based on some unique properties such as the well-defined and easy synthesis of the prodrug entities, near-quantitative assembly, and exceptionally high drug loading capacities, we predict that this platform has great potential for further clinical translations.

### Limitations of the study

DHA, an artemisinin derivative, is currently being investigated as an excellent anticancer agent beyond its established antimalarial activity.^32^ However, because of the high potency of cabazitaxel as a chemotherapeutic agent, we observed no cytotoxicity of DHA within the nanomolar concentration ranges. Therefore, the synergistic effects between DHA and cabazitaxel were not discussed in the present study.

## STAR★Methods

### Key resources table

**Table:** 

REAGENT or RESOURCE	SOURCE	IDENTIFIER
**Antibodies**		

Acetyl-alpha Tubulin (Lys40) Antibody	Affinity Biosciences	Cat#AF4351; RRID: AB_2844615
Alexa Fluor™ 555 donkey anti-Rabbit IgG (H+L)	ThermoFisher Scientific	Cat# A-31572; RRID: AB_162543

**Biological samples**		

Mouse tissues and organs	BALB/c nude mice	N/A

**Chemicals, peptides, and recombinant proteins**		

Cabazitaxel	Nanjing Jingzhu Biotechnology	Cat#Z100853; CAS: 183133-96-2
Artesunate	TCI	Cat#A2191; CAS: 88495-63-0
Dihydroartemisinin	Aladdin	Cat#D140839; CAS: 71939-50-9
3,3'-(Propane-2,2-diylbis(sulfanediyl))dipropionic acid	Tianjin Kailiqi Biopharma Technology	CAS: 4265-59-2
1,3-Diphenylisobenzofuran	Aladdin	Cat#D398432; CAS: 5471-63-6
1,2-Distearoyl-*sn*-glycero-3-phosphoethanolamine-*N*-[methoxy (polyethylene glycol) 2000	A.V.T	Cat#F01008; CAS: 147867-65-0
Hydrogen peroxide 30% aqueous solution	Sinopharm Chemical Reagent	Cat#10011218; CAS:7722-84-1
YF®488-Phalloidin	US EVERBRIGHT	Cat#YP0059S
DiI	US EVERBRIGHT	Cat#D4010; CAS:41085-99-8
DiR	US EVERBRIGHT	Cat#D4006; CAS:100068-60-8

**Critical commercial assays**		

Cell Counting Kit-8	MCE	Cat#HY-K0301
BeyoClick™ EdU Cell Proliferation Kit with Alexa Fluor 488	Beyotime	Cat#C0071S
Calcein-AM/PI Double Staining Kit	Dojindo	Cat#C542
Reactive Oxygen Species Assay Kit	Beyotime	Cat#S0033S

**Experimental models: Cell lines**		

Human: 143B cells	ATCC	Cat#CRL-8303
Human: U-2OS cells	ATCC	Cat#HTB-96
Human: Human Umbilical Vein Endothelial Cells (HUVEC)	ATCC	Cat#PCS-100-010
Mice: RAW264.7	The Cell Bank of Chinese Academy of Sciences	Cat#SCSP-5036

**Experimental models: Organisms/strains**		

BALB/c nude mice	Laboratory Animal Center of Hangzhou Medical College (Hangzhou, China)	N/A

**Software and algorithms**		

GraphPad Prism 9	GraphPad	https://www.graphpad.com/
Chemdraw 19.0	PerkinElmer	https://perkinelmerinformatics.com/products/research/chemdraw
FlowJo 10.0.7	BD Biosciences	https://www.flowjo.com/
MestReNova 14.0.0	Mestrelab Research S. L.	https://mestrelab.com/software/mnova/
Image J	National Institutes of Health	https://imagej.nih.gov/ij/download.html

**Others**		

High-Performance Liquid Chromatography, HPLC	Hitachi	Chromaster 5000; https://www.hitachi-hightech.com/
Zetasizer Nano ZS90	Malvern	Zetasizer Nano ZS90; https://www.malvernpanalytical.com/en/support/product-support/zetasizer-range/zetasizer-nano-range/zetasizer-nano-zs90
Nuclear Magnetic Resonance, NMR	BRUKER	AVANCE III 400Hz; https://www.bruker.com/
fluorescence Microscope	Olympus	IX71; https://www.fluorescencemicroscopes.com/olympus-model-ix71-fluorescence-microscope-phase-contrast/
UV-vis spectrometer	Shimadzu	UV-2700; https://www.shimadzu.com/an/products/molecular-spectroscopy/uv-vis/uv-vis-nir-spectroscopy/uv-2600i-uv-2700i/index.html

### Resource availability

#### Lead contact

Further information and requests for resources and reagents should be directed to and will be fulfilled by the Lead Contact, Shufeng Li (shufenglisd@126.com).

#### Materials availability

All data are available in the manuscript text and supplemental information. Reagents generated in this study will be made available on request, but we may require a payment and/or a completed Materials Transfer Agreement if there is potential for commercial application.

### Experimental model and study participant details

#### Cell culture

Human osteosarcoma 143B, U-2OS and K7 cells lines were obtained from ATCC. All cells were cultured in DMEM medium and maintained in atmosphere with 5% CO_2_ at 37°C. All culture media were supplemented with 10% fetal bovine serum (FBS), penicillin (100 units/mL) and streptomycin (100 μg/mL).

#### Mouse model of orthotopic osteosarcoma

All experimental BALB/c nude mice (4–5 weeks old, 18–20 g, male) were obtained from the Laboratory Animal Center of Hangzhou Medical College (Hangzhou, China). All mice were housed under a standard 12-hour light/ dark cycle with *ad libitum* food and water. The mouse studies were performed in accordance with the National Institute Guide for the Care and Use of Laboratory Animals. The Ethics Committee of the First Affiliated Hospital, Zhejiang University School of Medicine, approved the experimental protocols. An orthotopic 143B osteosarcoma model in nude mice was established to assess the *in vivo* biodistribution and therapeutic efficacy of CD nanoassemblies. For this purpose, 1×10^6^ 143B cells were implanted into the tibial bone marrow cavity of mouse to allow the tumor growth.

### Method details

#### Synthesis of diCTX

To a solution of cabazitaxel (350 mg, 0.42 mmol, 2.2 eq) in dry dichloromethane (DCM, 3 mL) was added 5,5-dimethyl-4,6-dithia-nonanedioic acid (48 mg, 0.19 mmol, 1.0 eq), 4-dimethylaminopyridine (DMAP) (76.7 mg, 0.63 mmol, 3.3 eq) and 1-(3-dimethylaminopropyl)-3-ethylcarbodiimide (EDC) (97.5 mg, 0.63 mmol, 3.3 eq). The reaction mixture was stirred at 45°C for 18 h. Upon the completion of reaction, the organic solvent was evaporated under vacuum. The residue was dissolved in DCM and washed with 5% aqueous citric acid, saturated aqueous NaHCO_3_ and brine. After the organic layer was dried over anhydrous Na_2_SO_4_, the residue was purified by flash column chromatography on silica gel (hexane: ethyl acetate = 2:1) to obtain the product (163.8 mg, 44.8%). The purity of diCTX was determined by the high-performance liquid chromatography (HPLC) to be 98.6%.

^1^H NMR (400 MHz, Chloroform-*d*) δ 8.11 (d, *J* = 7.7, 4H), 7.61 (t, *J* = 7.4, 2H), 7.50 (t, *J* = 7.6, 4H), 7.40 (m, 4H), 7.31 (m, 6H), 6.26 (br, 2H), 5.65 (d, *J* = 7.0, 2H), 5.46 (m, 4H), 5.35 (s, 2H), 5.00 (d, *J* = 9.5, 2H), 4.82 (s, 2H), 4.32 (d, *J* = 8.5, 2H), 4.17 (d, *J* = 8.4, 2H), 3.89 (dd, *J* = 10.7, 6.4, 2H), 3.84 (d, *J* = 6.9, 2H), 3.43 (s, 6H), 3.30 (s, 6H), 2.85–2.68 (m, 8H), 2.64 (m, 2H), 2.45 (s, 6H), 2.36–2.18 (m, 4H), 2.00 (s, 6H), 1.89–1.74 (m, 4H), 1.71 (s, 6H), 1.54 (s, 6H), 1.35 (s, 18H), 1.20 (m, 12H).

^13^C NMR (100 MHz, Chloroform-*d*): δ 205.07, 205.07, 171.11, 171.11, 169.77, 169.77, 168.22, 168.22, 167.04, 167.04, 155.22, 155.22, 139.55, 139.55, 137.27, 137.27, 135.01, 135.01, 133.65, 133.65, 130.20, 130.20, 130.20, 130.20, 129.25, 129.25, 128.96, 128.96, 128.96, 128.96, 128.68, 128.68, 128.68, 128.68, 128.28, 128.28, 126.41, 126.41, 126.41, 126.41, 84.18, 84.18, 82.51, 82.51, 81.57, 81.57, 80.69, 80.69, 80.53, 80.53, 78.85, 78.85, 76.47, 76.47, 74.74, 74.74, 74.68, 74.68, 72.16, 72.16, 57.16, 57.16, 57.12, 57.12, 56.81, 56.81, 56.45, 47.34, 47.34, 43.34, 43.34, 34.94, 34.94, 34.06, 34.06, 32.01, 32.01, 30.77, 30.77, 29.71, 29.71, 28.17, 28.17, 28.17, 28.17, 28.17, 28.17, 26.67, 26.67, 24.79, 24.79, 22.83, 22.83, 21.03, 21.03, 14.50, 14.50, 10.39, 10.39.

#### Synthesis of diDHA

To a solution of artesunate (270.4 mg, 0.70 mmol, 2.0 eq) in dry DCM (4 mL) was added dihydroartemisinin (100 mg, 0.35 mmol, 1.0 eq), DMAP (85.9 mg, 0.70 mmol, 2.0 eq) and EDC (109.2 mg, 0.70 mmol, 2.0 eq). The reaction mixture was stirred at 45°C for 18 h. Upon the completion of reaction, the organic solvent was evaporated under vacuum. The residue was dissolved in DCM and washed with 5% aqueous citric acid, saturated aqueous NaHCO_3_ and brine. After the organic layer was dried over anhydrous Na_2_SO_4_, the crude residue was purified by flash column chromatography on silica gel (DCM:methanol = 40:1) to afford diDHA (139.2 mg, 59.2%). The purity of diDHA was determined by HPLC to be 95.3%.

^1^H NMR (400 MHz, Chloroform-*d*) δ 5.78 (d, J = 9.8, 2H), 5.43 (s, 2H), 2.86–2.64 (m, 4H), 2.57 (m, 2H), 2.37 (td, J = 14.0, 3.9, 2H), 2.03 (m, 2H), 1.87 (m, 2H), 1.80–1.68 (m, 4H), 1.64–1.59 (m, 2H), 1.49 (m, 2H), 1.43 (s, 6H), 1.39 (d, J = 3.2, 1H), 1.35 (d, J = 3.3, 1H), 1.28 (m, 4H), 1.02 (m, 2H), 0.96 (d, J = 5.8, 6H), 0.86 (d, J = 7.1, 6H).

^13^C NMR (100 MHz, Chloroform-*d*) δ 171.06, 171.06, 104.45, 104.45, 92.16, 92.16, 91.47, 91.47, 80.10, 80.10, 53.54, 51.52, 45.20, 45.20, 37.25, 37.25, 36.19, 36.19, 34.07, 34.07, 31.77, 31.77, 28.83, 28.83, 25.96, 25.96, 24.57, 24.57, 21.97, 21.97, 20.23, 20.23, 12.08, 12.08.

#### Preparation and characterization of nanoassemblies

CD nanoassemblies coloaded with dimer cabazitaxel (diCTX) and dimer dihydroartemisinin (diDHA) were prepared using a one-step reprecipitation method. Briefly, diCTX (11.29 mg) and diDHA (7.79 mg) prodrugs were dissolved in 1 mL of dimethyl sulfoxide (DMSO) at a molar ratio of 1:2, and then the mixture solution was quickly injected into 9 mL of deionized (DI) water under ultrasonication. For *in vitro* and *in vivo* use, the nanoassembly solutions were dialyzed against DI water with a dialysis tubes (Spectrum, molecular weight cutoff of 7 kDa) for 24 h to remove DMSO. To PEGylate these nanoassemblies, the prodrug mixture and DSPE-PEG_2k_ were mixed at the mass ratio of 10:1 in 1 mL of DMSO, the mixture was injected into 9 mL of DI water and then dialyzed using the same method. Other nanoassemblies were prepared using the same protocol. Finally, the drug concentrations of nanoassemblies were determined by HPLC.

The size, zeta potential and polydispersity index of nanoassemblies were characterized at room temperature by dynamic light scattering (DLS) analysis (Zetasizer Nano ZS90, Malvern, UK). To characterize the morphology of CD nanoassemblies, the nanoparticle solution with 0.3 mg/mL cabazitaxel equivalent concentration was subjected to observation under transmission electron microscopy (TEM) at 120 kV acceleration voltage.

#### Stability test of CD nanoassemblies

The CD nanoassemblies (0.1 mg/mL, cabazitaxel equivalent concentration) was prepared in DI water, phosphate-buffered saline (PBS), or PBS containing 10% (v/v) FBS separately. The changes in particle size and PDI were measured by DLS for 7 days.

#### Molecular dynamics (MD) simulation

The chemical structures of both prodrugs were optimized, and the partial atomic charges were calculated using the Gaussian 09 software package. Force Field parameters were used for small molecules. Eighteen small molecules (the molar ratio of diCTX to diDHA = 1/2) were packed randomly by PACKMOL in a cubic box with a length of 100 Å. The mixture was then neutralized by the addition of sodium/chlorine counter ions and solvated in a cuboid box of TIP3P (transferable intermolecular potential 3P) water molecules with solvent layers 10 Å between the box edges and solute surface.

All MD simulations were performed using GROMACS. The particle mesh Ewald (PME) method was used to treat long-range electrostatic interactions. Before the production run, the systems were relaxed by 10000 steps using the conjugate gradient method. For the production run, an integration time step of 1 fs was employed to integrate the equations of motion. The simulated temperature was set to 300 K. In production phase, 100 ns simulation was carried out.

#### Encapsulation efficiency (EE) and drug loading content (DLC)

The EE and DLC in CD nanoassemblies were determined by HPLC. Briefly, CD nanoassemblies were prepared and centrifuged at 12,000 rpm for 10 min to remove unformulated dimeric prodrugs. Supernatant was subsequently recovered and diluted with acetonitrile to quantify the amounts of each drug. The EE and DLC of cabazitaxel or DHA in nanoassemblies were calculated as the following formulas:

EE (%) = W_cabazitaxel or DHA_ in nanoassemblies / W_feed_ × 100% (1)

DLC (%) = W_cabazitaxel or DHA_ in nanoassemblies / W_total_ × 100% (2)

#### Critical micelle concentration (CMC)

CD nanoassemblies-containing solutions ranging from 1.95 × 10^−4^ to 0.1 mg/mL (cabazitaxel equivalence) were prepared, and the scattering intensity of samples was determined by Malvern Nano ZS90 at 25°C. The CMC was calculated by plotting the scattering intensity as a function of the logarithm of concentration.

#### Reactive oxygen species (ROS) generation ability in aqueous solutions

The ROS generation ability of DHA with Fe^2+^ was detected by UV-vis spectrometer using 1,3-diphenylisobenzofuran (DPBF) as a ROS indicator. Briefly, Fe^2+^, DHA + Fe^2+^ solutions were added to DPBF solution. At predetermined timepoints of 10, 30, 60, 120, 240, 360 and 480 min, the absorbance of DPBF of each mixture was recorded using a UV-vis spectrometer, the DPBF absorption decrease was defined to reflect the generation rate of ROS. Blank DPBF solution was set as the control.

#### H_2_O_2_-triggered hydrolysis of the diCTX prodrug

The diCTX prodrug (0.1 mg/mL cabazitaxel equivalence) was incubated in 3 mL of acetonitrile/PBS (v/v, 7:3) solution with H_2_O_2_ at a variety of concentrations (0, 40, 80 mM), and the mixed solution was incubated at 37°C under shaking. At predetermined timepoints of 0.5, 1, 2, 4, 6, 8, 12, 24, 48, 72, and 96 h, 250 μL of the mixture was collected and diluted with 250 μL of acetonitrile and water (v/v, 1:1) for further analysis by HPLC.

#### *In vitro* cytotoxicity assay

The cell counting kit-8 (CCK-8) assay was applied to determine the *in vitro* cytotoxicities of free cabazitaxel, free DHA, free cabazitaxel plus DHA, diCTX nanoassemblies and CD nanoassemblies in osteosarcoma cell lines, while normal cells were incubated with free cabazitaxel plus DHA or CD nanoassemblies. Cells (1-5×10^3^ cells per well) were seeded into 96-well microplates and adhered for 24 h at 37°C. The fresh medium containing different concentrations of drugs were then added to cells and incubated for another 72 h. Cells incubated with fresh medium were used as control group. Following 72-h incubation with drugs, cell viability was evaluated using the CCK-8 assay and quantified by a microplate reader (Multiskan FC, Thermo Scientific) at 450 nm.

To compare the inhibitory effects of CD nanoassemblies with diCTX nanoassemblies, cells (1.3×10^3^ cells per well) were plated in 96-well plates and incubated with different concentrations of drugs. After treatment for 6 h, 12 h, or 24 h, the media containing drugs were abandoned and added with new fresh media to each well. After additional incubation for 66 h, 60 h, or 48 h, cell viability was determined by the CCK-8 assay.

#### Cellular uptake mechanism for CD nanoassemblies in 143B cells

To determine the cellular uptake of the CD nanoassemblies and whether the active transport dominates cellular uptake of CD nanossemblies, a lipophilic NIR probe DiI was encapsulated into the nanoparticles to construct DiI-labeled CD nanoassemblies, and the cellular uptake was examined by flow cytometry analysis. Briefly, 143B cells were seeded into six-well plates at a density of 3×10^5^ cells per well and cultured overnight. To examine whether the endocytosis was energy-dependent, 143B cells were incubated with DiI-labeled CD nanoassemblies at 37°C or 4°C for 4 h. Finally, the cells were harvested and washed with PBS, and the intracellular fluorescence was determined by flow cytometry analysis.

To investigate the exact pathways of cellular internalization for CD nanoassemblies, 143B cells were precultured in media containing specific endocytosis inhibitors chlorpromazine (10 μg/mL), cytochalasin D (40 μM), or filipin III (5 μg/mL) for 30 min. Subsequently, DiI-loaded CD nanoassemblies were added to cells and incubated for additional 4 h. Cells that incubated with fresh medium were regarded as negative controls, whereas cells that were not precultured with inhibitors were regarded as positive controls. Finally, the cells were harvested and washed with PBS, and the fluorescence intensities were measured by flow cytometry.

#### Intracellular ROS production

Intracellular ROS production was detected with the ROS fluorescence indicator 2′,7′dichlorofluorescein diacetate (DCFHDA). 143B cells were seeded into 12-well microplates at a density of 6×10^4^ cells per well and adhered overnight. To verify the ROS generation by DHA, cells were treated with free DHA, diDHA nanoassemblies, diCTX nanoassemblies and CD nanoassemblies at the same DHA concentration (360 nM) at 37°C for 24 h. After the incubation with drugs, cells were washed with cold PBS and incubated with DCFHDA (10 μM) for another 20–30 min in the dark. Finally, the intracellular fluorescence of DCF were observed under fluorescence microscopy at 488 nm.

#### Cell proliferation study by the EdU test

143B cells were seeded into flat-bottomed 12-well plates with 8×10^4^ cells per well. After incubated at 37°C for 24 h, different drugs containing with the same cabazitaxel equivalent concentration (30 nM) were then added to the cells and incubated for an additional 48 h at 37°C. After drug treatment, cell proliferation was quantified *via* BeyoClick™ EdU Cell Proliferation Kit (Shanghai, China) according to the manufacturer’s protocol. Briefly, 5-ethynyl-2′-deoxyuridine (EdU), a thymidine analog, which is enable to replace thymine (T) during DNA replication, was added into each individual well and incubated for 2 h at 37°C. Next, 4% formaldehyde was added to fix the cells for 20 min at room temperature, and then the cells were incubated with 300 μL of 0.5% Triton X-100 for 15 min. After that, the cells was treated with azide-labeled Alexa Fluor 488 for 30 min, and the nuclei were stained with Hoechst 33342 for 10 min in the dark. Finally, the cells were washed with PBS three times and imaged using fluorescence microscope (Olympus, IX71).

#### Calcein-AM/propidium iodide (PI) staining assay

To evaluate the synergistic effect of CD nanoassemblies intuitively, Calcein-AM staining was employed to label live cells while PI stained dead cells. Briefly, 143B cells (4×10^4^ cells per well) were seeded into 12-well microplates and cultured overnight. Different drugs containing with the same cabazitaxel-equivalent concentration (240 nM) were then added to each well and incubated for 24 h. After wash with cold PBS, the cells were co-stained with calcein-AM/PI at the working concentration for 30 min in the dark according to the manufacturer’s protocol. After brief wash, the cells were observed on fluorescence microscopy.

#### Microtubule bundle formation analysis

To explore whether the observed cytotoxicity is associated with released cabazitaxel, the immunofluorescence staining of tubulin was performed. Briefly, 143B cells were seeded into a glass-bottom dish at the density of 4×10^4^ cells per well and cultured overnight. After 48-h incubation with free cabazitaxel (30 nM) plus DHA (60 nM) or CD nanoassemblies (30 nM cabazitaxel equivalent concentration and 60 nM DHA equivalent concentration), cells were fixed with 4% paraformaldehyde for 30 min. Subsequently, the cells were permeated with 0.5% Triton X-100 for 2 h at room temperature and blocked with 5% bovine serum albumin for 1 h. The cells were immunostained with acetyl-alpha tubulin antibody at 4°C overnight and further incubated with Alexa Fluor 555 donkey anti-rabbit IgG for 1 h and Alexa Fluor 488-phalloidin for 40 min. Finally, the cell nuclei were stained with 4′,6-diamidino-2-phenylindole (DAPI) for 10 min and observed on fluorescence microscope (Olympus, IX71).

#### *In vivo* biodistribution in the orthotopic osteosarcoma model

An orthotopic 143B osteosarcoma model in BALB/c nude mice was established to investigate the *in vivo* biodistribution of CD nanoassemblies. Briefly, 1×10^6^ 143B cells were implanted into the tibial bone marrow cavity of each mouse. When the tumor grew to 200–300 mm^3^ in volume, mice were randomly divided into two groups (n = 4 per group) and intravenously injected with free DiR or DiR-labeled CD nanoassemblies at the DiR equivalent dose of 1 mg/kg (free DiR was prepared in polysorbate 80/ethanol,1:1, v/v). At 24 h post-administration, mice were sacrificed. Major organs (e.g., heart, liver, spleen, lung, and kidney) and tumors were dissected for *ex vivo* imaging at Clairvivo OPT (SHIMADZU Corporation, Kyoto, Japan). To analyze the drug accumulation in tumors, the tumor tissues were prepared as frozen sections and stained with DAPI. Frozen sections were imaged by CLSM (FV3000, Olympus, Japan).

#### *In vivo* antitumor efficacy against the osteosarcoma model

To evaluate the antitumor efficacy of CD nanoassemblies, orthotopic 143B osteosarcoma model was established and given treatment. When the tumors reached approximately 100 mm^3^ in volume, mice were randomly divided into three groups (n = 7 per group). The animals were intravenously administered with saline, free drug combination (cabazitaxel plus DHA), and CD nanoassemblies (at cabazitaxel equivalent dose of 7.0 mg/kg) through the tail vein on days 0, 3, and 6. Free cabazitaxel was prepared in polysorbate 80/ethanol (1:1, v/v) and injected via the tail vein, while free DHA (4.8 mg/kg) was dissolved in DMSO and administered by intraperitoneal injection. Tumor volumes and body weights were monitored every three days, and the tumors were photographed at the same time. The formula tumor volume (V) = (L × W^2^)/2 (L: length, W: width, W is smaller than L) was used to calculate tumor volume.

#### Histological analysis

At the end of treatment, major organs and tumors were fixed with 4% formaldehyde, embedded in paraffin, sectioned into 5 μm and stained with hematoxylin and eosin (H&E). In addition, the fixed tumor tissues were stained by the *In-Situ* Cell Death Detection Kit (Fluorescein, Roche Applied Science) to detect DNA fragmentation by the terminal deoxynucleotidyl transferase (TdT)-mediated dUTP nick end labeling (TUNEL) apoptosis assay according to the manufacturer’s protocol. The stained slices were imaged on fluorescence microscope (Olympus, IX71).

### Quantification and statistical analysis

All quantitative data are presented as the mean ± standard deviation (SD). Student’s *t* test or one-way ANOVA test were applied to estimate statistical significance between measurements. Statistical significance was indicated by p values <0.05 (∗), <0.01 (∗∗), <0.001 (∗∗∗), or <0.0001 (∗∗∗∗). All the statistical analyses were conducted with GraphPad Prism 9 (GraphPad software).

## Data Availability

• Data reported in this paper will be shared by the lead contact upon reasonable request. • This paper does not report any original code. • Any additional information required to reanalyze the data reported in this paper is available from the lead contact upon request.
