# Supplementary material for: Dynamic nanoassemblies derived from small-molecule homodimeric prodrugs for in situ drug activation and safe osteosarcoma treatment
Source: iScience. 2023 Jul 17;26(8):107409. doi: 10.1016/j.isci.2023.107409 (PMC10404730; doi:10.1016/j.isci.2023.107409)
Supplement: Document S1. Figures S1–S8 and Tables S1 and S2 [file mmc1.pdf]

iScience, Volume *26*

Supplemental information

Dynamic nanoassemblies derived from small-molecule homodimeric prodrugs for *in situ* drug activation and safe osteosarcoma treatment

Jian Wang, Peirong Xu, Yeyong Zhang, Shuai Han, Gongteng Wang, Hangxiang Wang, Haihan Song, and Shufeng Li

Supplementary Scheme S1 (Related to Figure 1): Synthesis of diCTX.

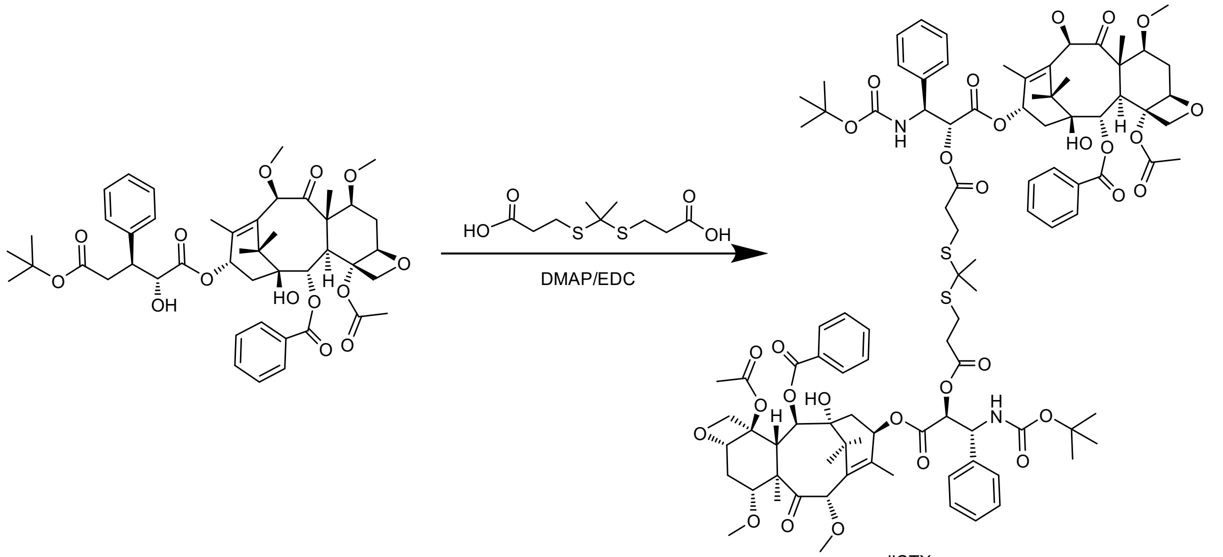

diCTX

Synthetic scheme of diCTX.

Supplementary Figure S1 (Related to Figure 1): ^{1}H NMR characterization of diCTX.

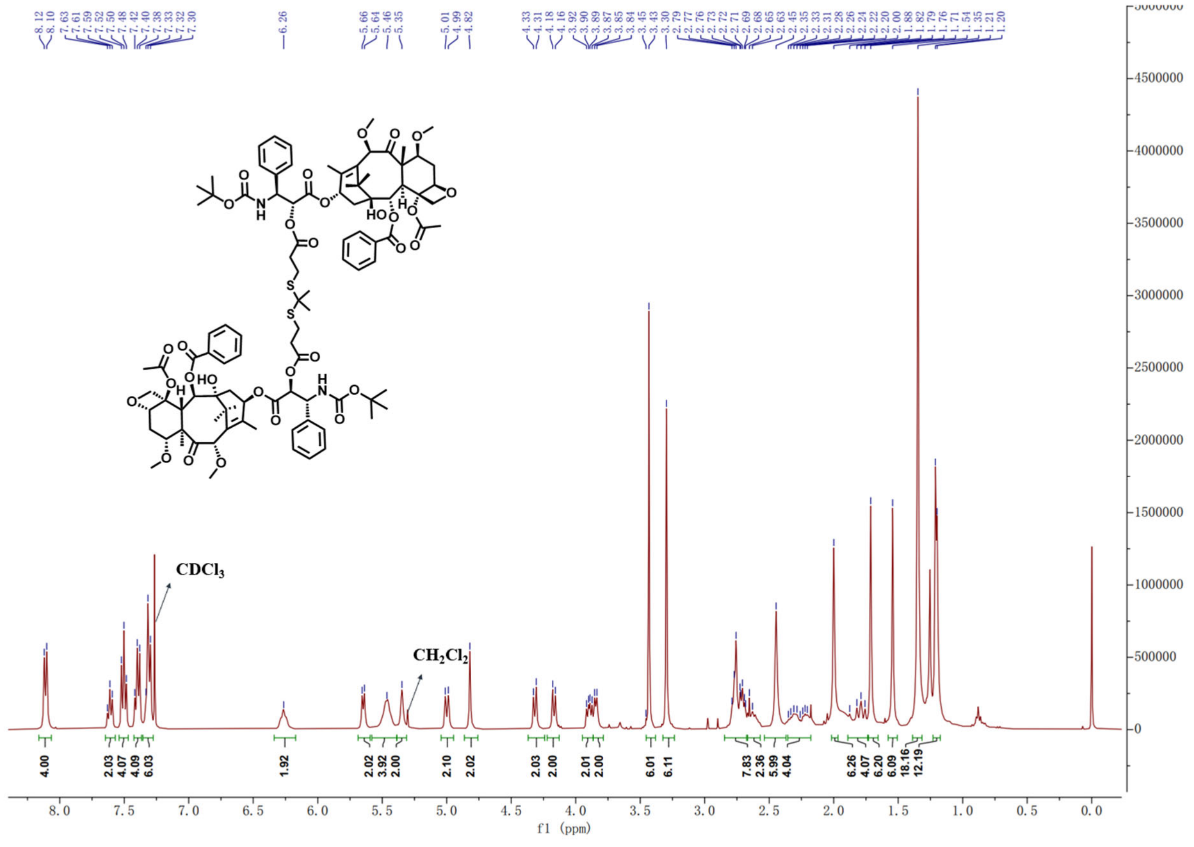

^{1}H NMR spectrum of diCTX in $CDCl_3$.

Supplementary Figure S2 (Related to Figure 1): ^{13}C NMR characterization of diCTX.

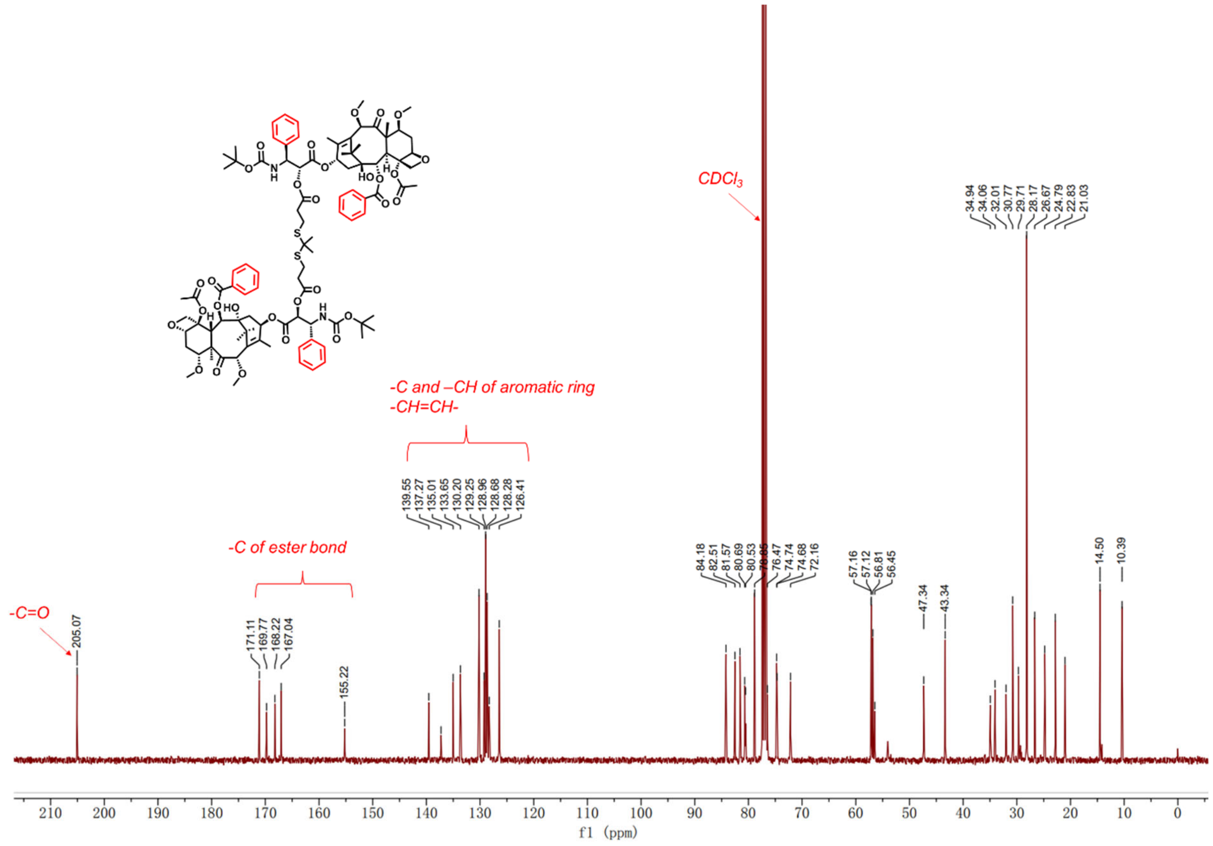

^{13}C NMR spectrum of diCTX in $CDCl_3$.

Supplementary Scheme S2 (Related to Figure 1): Synthesis of diDHA.

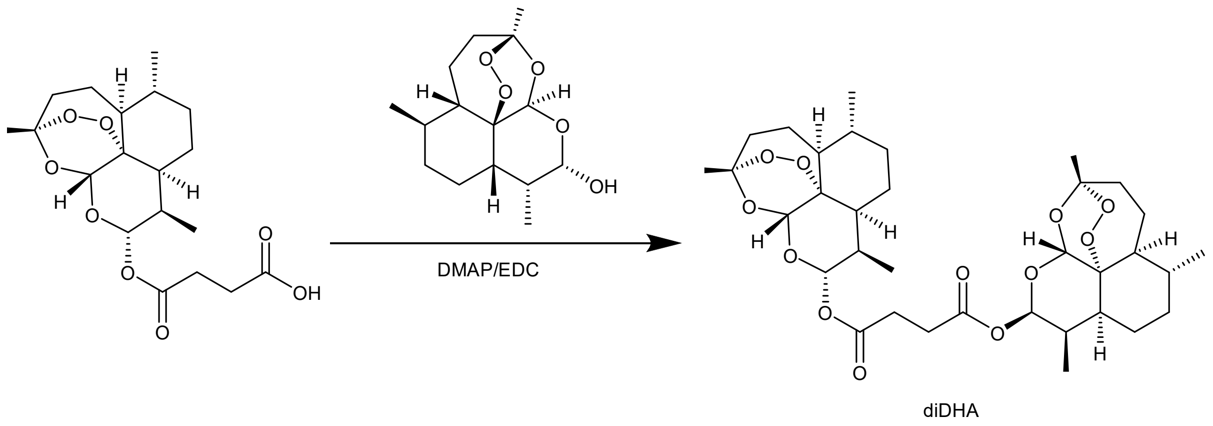

Synthetic scheme of diDHA.

Supplementary Figure S3 (Related to Figure 1): ^{1}H NMR characterization of diDHA.

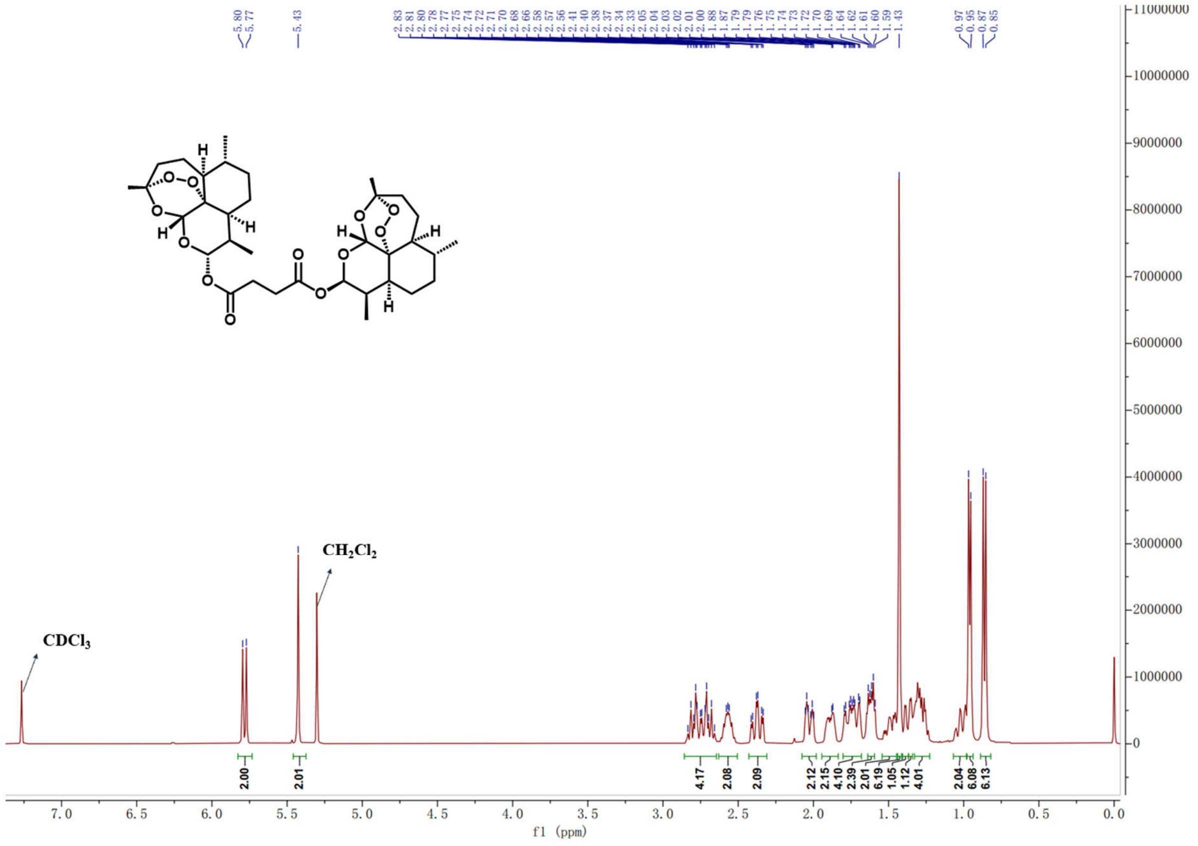

^{1}H NMR spectrum of diDHA in $CDCl_3$

Supplementary Figure S4 (Related to Figure 1): ^{13}C NMR characterization of diDHA.

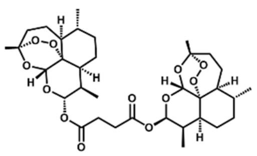

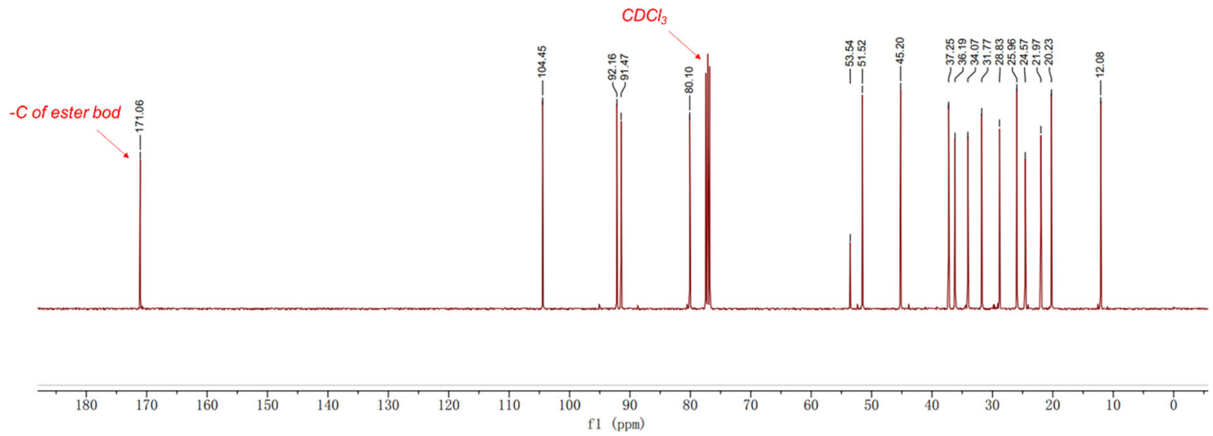

^{13}C NMR spectrum of diDHA in $CDCl_3$.

Supplementary Figure S5 (Related to Figure 1): Purity characterization of diCTX and diDHA by HPLC.

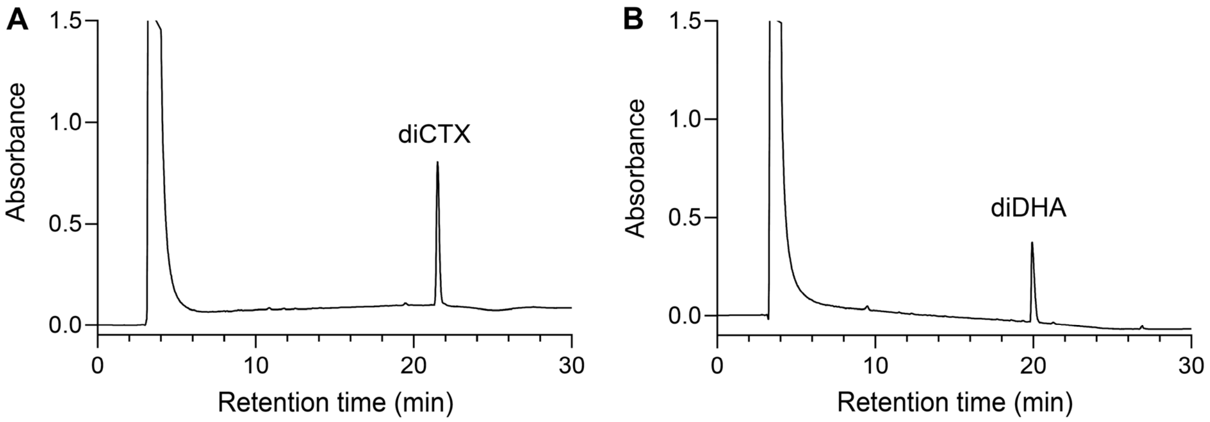

HPLC analysis of diCTX (A) and diDHA (B) prodrugs. diCTX and diDHA were subjected to analytical HPLC using a C8 YMC-Pack ODS column (5 μm, 250 × 4.6 mml). A gradient of 30-100% acetonitrile in water within 35 min was adopted as the mobile phase at a flow rate of 1 mL/min. UV detection was at a wavelength of 220 nm.

Supplementary Figure S6 (Related to Figure 2): Characterization of self-assembly capability of chemically unmodified DHA.

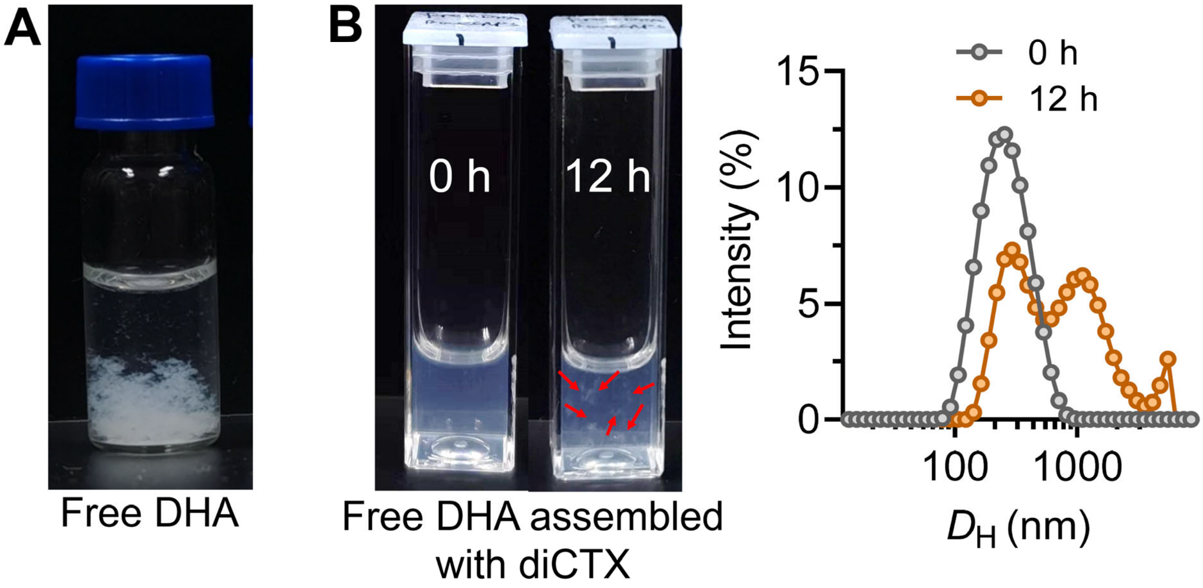

(A) Photograph of the solutions of free DHA following the nanoprecipitation protocol. (B) Photographs of the particle solutions after 0 h and 12 h storage (left). Variations of size distribution of nanoassemblies analyzed by DLS (right).

Supplementary Figure S7 (Related to Figure 3): Characterization of bare coassembly prepared from diCTX and diDHA prodrugs.

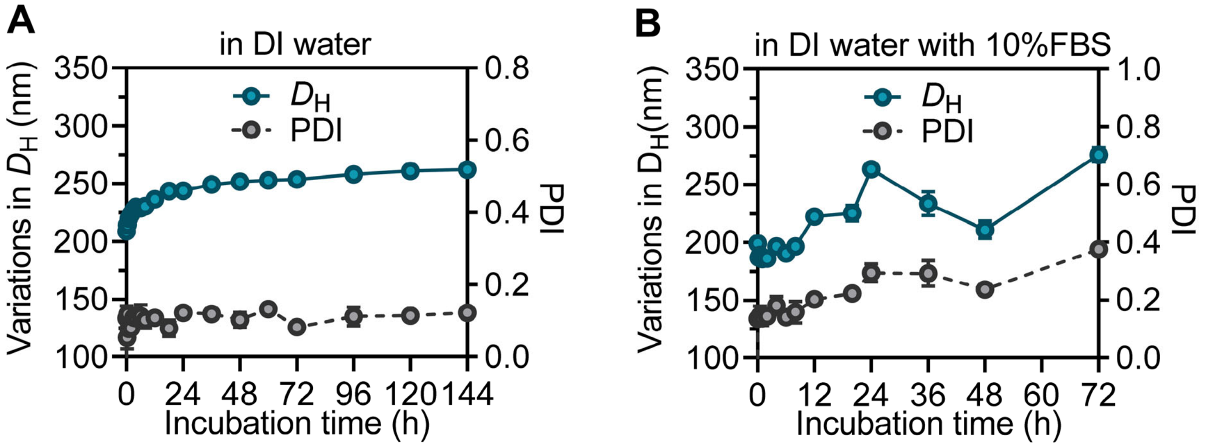

Particle size and PDI measurements of diCTX- and diDHA-assembled nanoparticles in DI water (A) and DI water containing 10% (v/v) fetal bovine serum (B), as monitored by changes in particle sizes and PDI.

Supplementary Figure S8 (Related to Figure 5): *In vitro* cytotoxicity of drugs in normal cells.

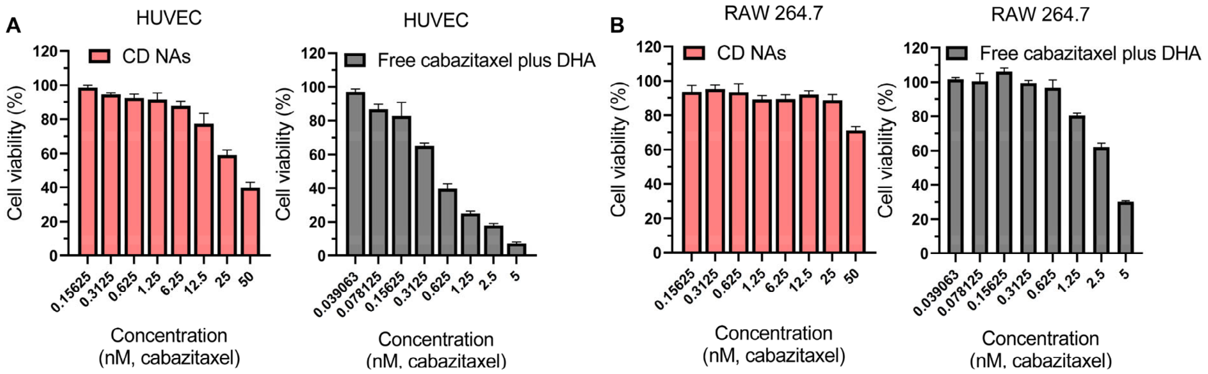

In vitro cytotoxicity of drugs in HUVEC (A) and Raw 264.7 (B). The cells were exposed to drugs for 72 h, and cell viability was determined by the CCK-8 assay. Data are presented as the mean ± SD (n = 3).

Supplementary Table S1 (Related to Figure 2): The encapsulation efficiency (EE) and drug loading content (DLC) for CD nanoassemblies (NAs).

Drug formulations	Drugs	EE (%)[a]	DLC (%) [b]
CD NAs (Cabazitaxel/DHA = 1:2, molar ratio)	Cabazitaxel	99.0±0.5	52.3±0.2
	DHA	99.2±0.5	35.6±0.2
	Cabazitaxel + DHA	-	88.0±0.4

[a] EE: Drug encapsulation efficiency.
[b] DLC: Drug loading content.

Supplementary Table S2 (Related to Figure 5): IC_{50} values extrapolated from dose-response curves shown in Figure 5A and Figure S8. Cells were treated with free drug combination or CD nanoassemblies (NAs), and the cell viability was determined by the CCK-8 assay. The data are presented as the means ± SD (n = 3).

**Drug formulations**	**IC_{50} (nM)**				
	**Cancer cells**			**Normal cells**	
	**143B**	**U-2OS**	**K7**	**RAW 264.7**	**HUVEC**
Free cabazitaxel plus DHA	1.4±0.4	0.5±0.2	0.6±0.05	3.1±0.1	0.5±0.02
CD NAs (Fold increase)[a]	35.2±3.8 (25.1)	13.8±4.1 (27.6)	15.0±1.4 (25.0)	104.8±7.0 (33.6)	36.9±1.7 (72.5)

[a] Fold increase: IC_{50}(CD NAs) / IC_{50}(Free cabazitaxel plus DHA);
